# Cascade Promotion of Gas Separation Performances in CMS Membranes: MOFs With Functional Groups and Loaded Noble Metals

**DOI:** 10.1002/advs.202503471

**Published:** 2025-06-05

**Authors:** Min Deng, Jing Wei, Jundong Guo, Zikang Qin, Jia Song, Junfeng Zheng, Lin Yang, Lu Yao, Wenju Jiang, Xiaohua Ma, Xuezhong He, Jiadai He, Jianjian Wang, Zhongde Dai

**Affiliations:** ^1^ College of Architecture and Environment Sichuan University Chengdu 610065 P. R. China; ^2^ National Engineering Research Centre for Flue Gas Desulfurization Chengdu 610065 P. R. China; ^3^ Carbon Neutral Technology Innovation Center of Sichuan Chengdu 610065 P. R. China; ^4^ College of Carbon Neutrality Future Technology Sichuan University Chengdu 610065 P. R. China; ^5^ DongFang Boiler Co., Ltd. Zigong Sichuan 643001 P. R. China; ^6^ State Key Laboratory of Separation Membranes and Membrane Processes School of Materials Science and Engineering Tiangong University Tianjin 300387 P. R. China; ^7^ Department of Chemical Engineering Guangdong Technion‐Israel Institute of Technology 241 Daxue Road Shantou Guangdong 515063 P. R. China; ^8^ School of Chemistry and Chemical Engineering Chongqing University Chongqing 401331 P. R. China

**Keywords:** carbon molecular sieve membranes, CO_2_ capture, H_2_ separation, membrane gas separation, nanofiller

## Abstract

The rational design of precursor structure serves as a critical determinant for the pore geometry and gas separation performance of carbon molecular sieve (CMS) membranes. Herein, a novel mixed‐matrix CMS (MMCMS) membrane was fabricated via a palladium‐doped carboxyl‐functionalized UiO‐66 (Pd/UiO66‐COOH)/polyimide (PI) MMM precursor. On one hand, the decomposition of ‐COOH groups generates abundant micropores, meanwhile the decarboxylation‐induced thermally cross‐linking enhances the stability of the carbon framework, thus mitigating the collapse of micropores during carbonization and consequently improving H_2_ and CO_2_ permeability, as well as membrane stability; on the other hand, the synergistic effect of decarboxylation‐induced thermally cross‐linking and the catalytic graphitization effects of Pd nanoparticles facilitated the formation of more ordered Langmuir domains and narrowed carbon interlayer spacing, thereby enhancing molecular sieving effects. In addition, Pd nanoparticles also contribute to providing abundant H_2_ adsorption sites to facilitated transport of H_2_ gases. Specifically, the PI/Pd‐UiO66‐COOH‐5‐550 MMCMS membrane exhibited superior H_2_ permeability of 9134.6 Barrer (P_CO2_ = 4033.4 Barrer) with H_2_/CH_4_ selectivity of 118.5 (α_CO2/CH4_ = 52.3), exceeding the latest Robeson upper bound. Furthermore, the membrane also demonstrated attractive aging resistance, retaining over 90% of its initial H_2_ and CO_2_ permeability after a 21‐day long‐term stability test.

## Graphical abstract

Novel mixed‐matrix carbon molecular sieve (MMCMS) membranes are fabricated via employing palladium‐doped carboxyl‐functionalized UiO‐66 (Pd/UiO66‐COOH)/polyimide MMM as precursor. The synergistic effect of decarboxylation‐induced thermally cross‐linking and catalytic graphitization of Pd promoted the formation of more ordered Langmuir domains, thereby enhancing gas separation performance. Pd nanoparticles also contributed to providing abundant adsorption sites to facilitate H_2_ gas transport.

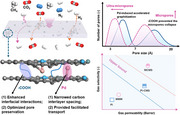

## 1 Introduction

H_2_ separation/purification and CO_2_ capture are key strategies in tackling the dual challenges of energy shortages and environmental degradation, playing an important role in the transition to a carbon‐neutral society.^[^ ^1^, ^2^ ^]^ Membrane separation technology serves as an effective alternative to traditional separation methods,^[^ ^3^ ^]^ offering advantages including small footprint, high scalability, environmental friendliness, and low energy consumption.^[^ ^4^, ^5^, ^6^ ^]^ Nevertheless, there is a trade‐off between permeability and selectivity in most studied polymer membranes, refined as Robeson upper bound.^[^ ^7^ ^]^ Carbon molecular sieve (CMS) membranes, obtained through the high‐temperature carbonization of precursor membranes,^[^ ^8^, ^9^ ^]^ possess tunable pore structures, exceptional thermochemical stability, as well as attractive gas separation performance, which have garnered extensive research interest.^[^ ^10^, ^11^ ^]^

Generally, during the pyrolysis of CMS membranes, the isolated strands and fused fragments with analogous structure align and fuse.^[^ ^12^ ^]^ A portion of the movable carbon chains organizes into more ordered “graphene‐like” plates, designated as Langmuir domains (“L”), which correspond to a significant abundance of ultra‐micropore structures (<7 Å) and exhibit a strong sieving effect. The remaining carbon chains are randomly packed, providing a disordered continuous phase (“C”) that contains micropore structures (<20 Å), thereby enabling a high permeability. The distribution and population of ultra‐micropores/micropores are critical factors governing gas transport and molecular sieving in CMS membranes. Through rational precursor design, pore structures can be precisely optimized, leading to substantial gas separation performance enhancement.^[^ ^13^, ^14^, ^15^, ^16^ ^]^

Research has demonstrated that the incorporation of porous fillers into precursor membranes for the fabrication of mixed matrix membranes (MMMs) not only effectively regulates the ultra‐microporosity, but also induces the formation of additional micropores in CMS membranes, improving gas permeation performance.^[^ ^17^ ^]^ Bae et al. fabricated mixed‐matrix CMS (MMCMS) membranes for CO_2_/N_2_ separation by incorporating SAPO‐34 zeolite as a filler into polyimide (PI) matrix. The introduction of SAPO‐34 simultaneously enhanced both CO_2_ permeability (from 1126 Barrer to 2615 Barrer) and CO_2_/N_2_ selectivity (from 19.3 to 31.7).^[^ ^18^ ^]^ Metal–organic frameworks (MOFs) were strategically incorporated as fillers to engineer high‐performance MMCMS membranes as well. ZIF‐8 was introduced into the phenolphthalein‐based cardo poly (arylene ether ketone) (PEK‐C) to prepare MMCMS membranes.^[^ ^20^ ^]^ The results demonstrated that the presence of ZIF‐8 nanoparticles resulted in a 1.8 times higher CO_2_ permeability (8902 Barrer) compared to the neat PEK‐C CMS membrane. In addition, UiO‐66, exhibits superior chemical/thermal stability, high specific surface area and porosity, as well as the advantages of functional modification,^[^ ^21^ ^]^ enables it to serve as an ideal porous filler for CO_2_ separation in MMMs.^[^ ^22^ ^]^ The incorporation of UiO‐66‐NH_2_ into PI matrices has been demonstrated to modulate the ultra‐microporous structure of CMS membranes,^[^ ^19^ ^]^ specially, narrowed bimodal pore size distribution in the ultra‐microporous region, thereby effectively facilitated their molecular sieving performance.

On the other hand, incorporating metal into the precursors to prepare CMS membranes has also been proven to be an effective method regulate the pore geometry in CMS membranes, thus enhancing molecular sieving.^[^ ^23^, ^24^ ^]^ Wang et al. prepared PI‐CMS membranes containing Zn^2+^, and the Zn^2+^ functionalization significantly enhanced the selectivity of the CMS membranes (H_2_/CH_4_ selectivity increased from 84.3 to 370), along with a slight increase in H_2_ permeability (from 5852 Barrer to 6768 Barrer).^[^ ^25^ ^]^ Recently, Pd nanoparticles were anchored in the carbon strands of CMS membranes, inducing the arrangement of carbon matrix into a more ordered structure and promoting the formation of ultra‐micropores (<3.3 Å) within the CMS membranes, thereby enabling precise molecular sieving of H_2_.^[^ ^26^ ^]^

These findings revealed that incorporating metallic/porous nanofillers could effectively tailor the pore geometry of CMS membranes, leading to a substantial enhancement of gas separation performances. However, the inherent compatibility issue between the nano fillers and polymer matrices often induces non‐selective voids or defects, losing their molecular sieving effect and reducing the separation selectivity.^[^ ^27^, ^28^ ^]^

Furthermore, the 6FDA (4,4′‐hexafluoroisopropylidene diphthalic anhydride)‐based PI demonstrates enhanced gas permeability in CMS membranes,^[^ ^29^, ^30^ ^]^ because the bulky ‐CF_3_ groups in the 6FDA moiety can create steric hindrance between polymer chains, effectively inhibiting chain stacking and increasing the fractional free volume.^[^ ^31^ ^]^ Therefore, in the current work, with the aim of improving the nanofiller compatibility and regulate the pore geometry in the CMS membranes, UiO‐66 nanoparticles were first functionalized with ‐COOH groups, and then Pd nanoparticles (>1 wt.%) were loaded on the UiO‐66‐COOH surfaces by an excessive impregnation method (Pd/UiO66‐COOH) (Table S1, Supporting Information), the obtained functionalized nanofillers were added into 6FDA/TMPD PI matrix, MMCMS membranes were developed under different carbonization conditions. The physicochemical properties of the MMCMS membranes were systematically characterized using XRD, Raman, XPS, SEM and TEM. The results revealed that Pd nanoparticles tightened interior carbon chains, inducing an ordered arrangement of carbon structures and enhancing ultra‐microporosity, which resulted in an enhancement of H_2_/CH_4_ selectivity. Simultaneously, the decomposition of ‐COOH groups generated abundant micropores, meanwhile the decarboxylation‐induced thermally cross‐linking enhanced the stability of the carbon framework, suppressing the collapse of micropores during the carbonization process and consequently enhancing the H_2_ permeability and membrane stability. This strategy of dual‐functional integration of metal nanoparticles and cross‐linkable functional groups provides an effective pathway for designing high performance MMCMS membranes.

## 2 Results and Discussion

### 2.1 Precursor Membrane Fabrication and Characterization

**Figure 1:**
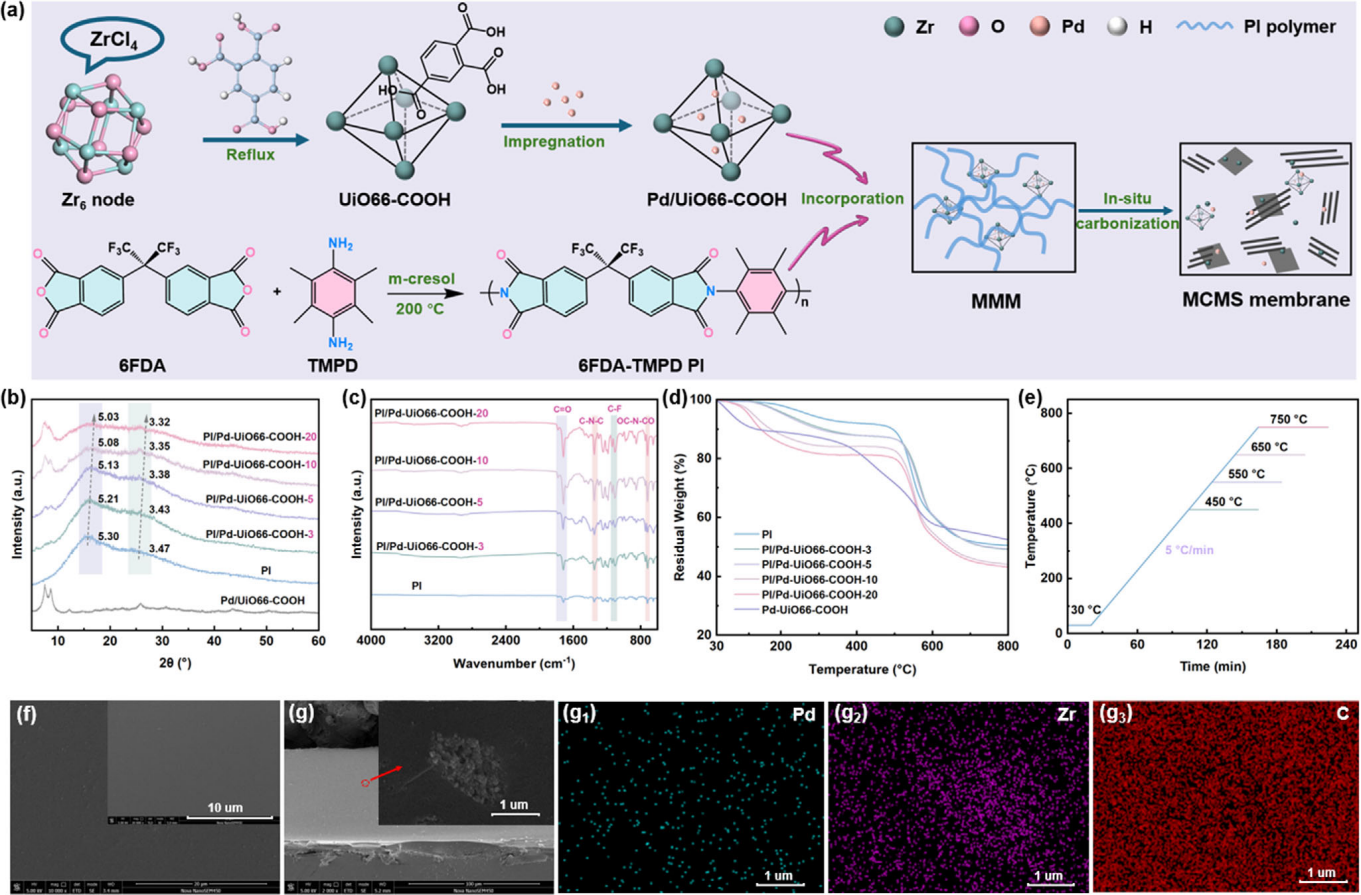
a) Schematic illustration of the MMCMS membrane preparation procedure; b) XRD pattern, c) FTIR spectra and d) TGA curve of PI/Pd‐UiO66‐COOH‐X precursor membranes; e) Carbonization protocols of CMS membranes; f) SEM surface image of PI/Pd‐UiO66‐COOH‐5‐550 precursor membranes (insert: corresponding MMCMS membrane); g) SEM cross‐section image (insert: SEM image of magnified partial of the selected region, and g1–g3) EDS mapping of magnified partial of the selected region) of PI/Pd‐UiO66‐COOH‐5‐550 MMCMS membrane.

The scheme of MMCMS membrane fabrication was shown in **Figure** **1**a, detailed experimental information could be found in Supporting Information. Through our previous systematic characterization (XRD, FTIR, TEM, etc.), it was found that the modification of ‐COOH and Pd had no significant change in the microstructure of UiO‐66.^[^ ^32^ ^]^ Therefore, this work focused on conducting a detailed investigation into the microstructure of Pd/UiO66‐COOH‐prepared PI/Pd‐UiO66‐COOH precursor membranes and their derived MMCMS membranes. The microstructures of the Pd/UiO66‐COOH and PI/Pd‐UiO66‐COOH‐X precursor membranes were characterized through XRD analysis. As displayed in Figure 1b, the PI/Pd‐UiO66‐COOH‐X membranes exhibited two diffraction signatures (2*θ* = 7–9°) consistent with the Pd/UiO66‐COOH. In addition, two broad peaks around 16° and 25° was observed, indicative of dual interchain spacings within the pristine PI membrane. Moreover, compared to the pristine PI, it was observed that the PI/Pd‐UiO66‐COOH‐X membranes exhibited a smaller d‐spacing. As the content of Pd/UiO66‐COOH increased, the d‐spacing decreased progressively from 5.30 to 5.03 Å (Table S2, Supporting Information), this reduction was due to the interactions between Pd/UiO66‐COOH and PI, which promoted a more compact arrangement of the polymer chains.^[^ ^33^, ^34^ ^]^

FTIR was employed to examine the chemical structure of the precursor PI/Pd‐UiO66‐COOH‐X membranes, and the results were presented in Figure 1c and Table S3 (Supporting Information). Specifically, the characteristic bands at about 1789 and 1720 cm^−1^ were attributed to the symmetric and asymmetric stretching vibrations of the C = O bonds in the imide ring, respectively. The bands at 1375 and 721 cm^−1^ were ascribed to the C ‐N ‐ C stretching vibration and the OC‐N‐CO imide ring band deformation, respectively.^[^ ^35^ ^]^ Notably, the peaks intensity of the precursor membranes increased with the increasing content of Pd/UiO66‐COOH, which could be explained by the interaction between the ‐COOH groups in Pd/UiO66‐COOH and the C = O or ‐NH‐ groups in the PI.

The thermal decomposition behavior of PI/Pd‐UiO66‐COOH membranes was analyzed using TGA (Figure 1d), a clear two stage decomposition curve could be found. Notably, the incorporation of Pd/UiO66‐COOH resulted in increased mass loss between 200–450 °C, which was likely attributed to the decarboxylation of ‐COOH groups.^[^ ^30^, ^36^ ^]^ In addition, a second stage from 450 to 600 °C, which may be due to decomposition of PI polymer chain.^[^ ^37^ ^]^ Based on the TGA results, the membrane carbonization temperatures were selected as 450, 550, 650, and 750 °C, with the detailed carbonization procedure shown in Figure 1e.

The morphology and structural changes of the PI and PI/Pd‐UiO66‐COOH‐X membranes before and after carbonization were investigated using SEM (Figure 1f; Figure S2, Supporting Information). It could be observed that both the PI and PI/Pd‐UiO66‐COOH‐X precursor membranes displayed a smooth, flat and dense defect‐free surface. In addition, as shown in Figure 1f and Figure S3 (Supporting Information), similar to the surface images of the precursor membranes, the CMS membranes inherited their microstructure. When the content of Pd/UiO66‐COOH reached 20 wt.%, it aggregated within the CMS membranes, and this phenomenon was also observed in the cross‐section images of the CMS membranes. Furthermore, the EDS mapping analysis of the cross‐section (Figure 1g; Figure S4, Supporting Information) revealed a uniform distribution of C elements within the membrane. Additionally, it was found that the Pd and Zr elements, apart from being distributed on the UiO66‐COOH framework, were also uniformly dispersed within the carbon matrix.^[^ ^38^ ^]^ This suggested that partial decomposition of Pd/UiO66‐COOH during the carbonization process, arising from the migration of metal elements, which in turn facilitated the development of well‐ordered microporous structure within the CMS membranes.

### 2.2 MMCMS Membrane Characterization

**Figure 2:**
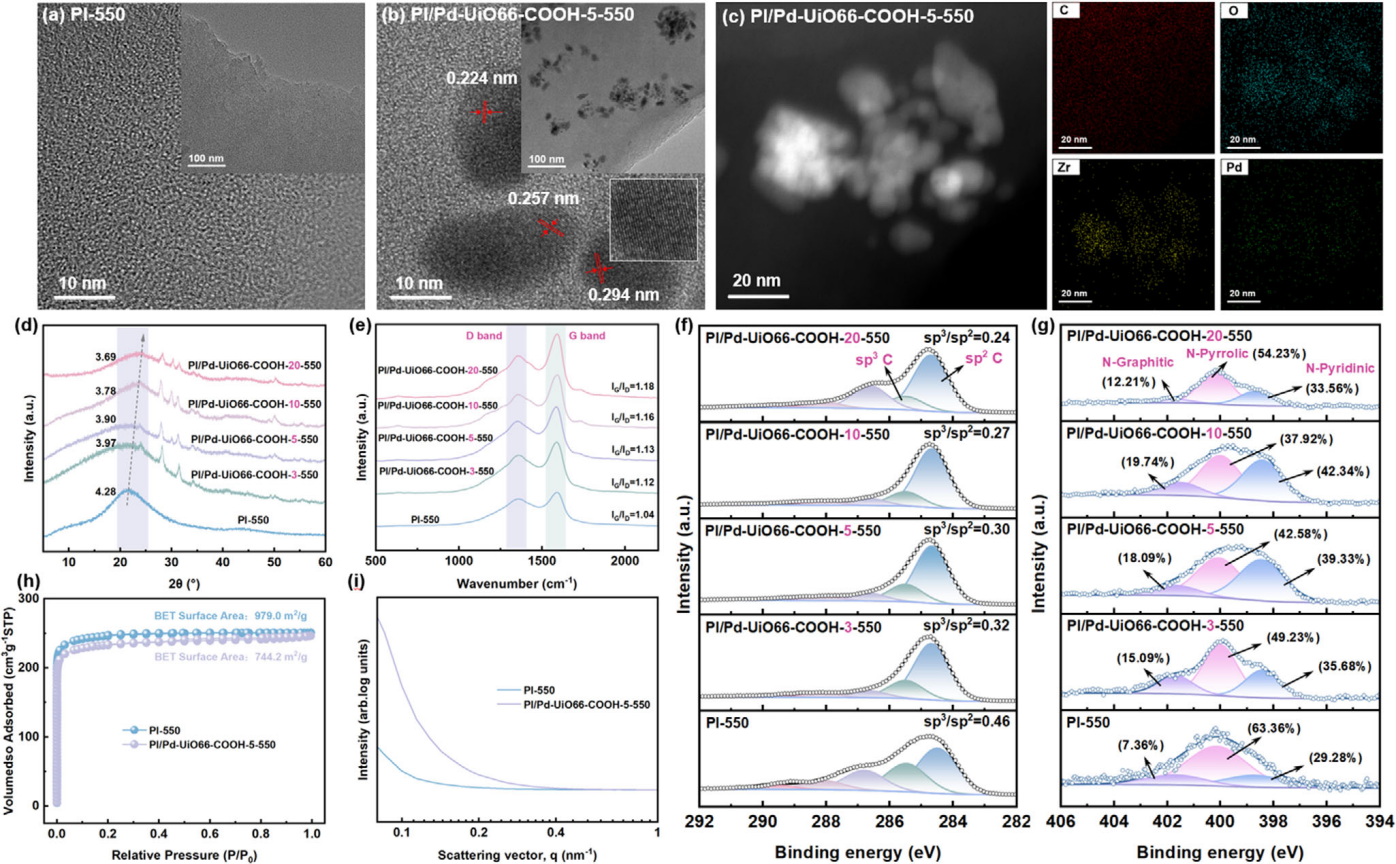
HRTEM image of a) PI‐550 CMS membrane and b) PI/Pd‐UiO66‐COOH‐5‐550 MMCMS membrane (insert: TEM images); c) HAADF‐STEM image of PI/Pd‐UiO66‐COOH‐5‐550 MMCMS and corresponding EDS mappings; d) XRD patterns, e) Raman curve, High‐resolution f) C1s and g) N1s XPS spectra of PI/Pd‐UiO66‐COOH‐X‐550 MMCMS membranes; h) N_2_ adsorption–desorption isotherms and i) SAXS intensity profiles of pristine PI‐550 CMS and PI/Pd‐UiO66‐COOH‐5‐550 MMCMS membranes.

The obtained MMCMS membranes were further characterized using various techniques. TEM results revealed that the CMS made from pristine PI membrane were amorphous (**Figure** **2**a), while the MMCMS made from PI/Pd‐UiO66‐COOH, metal particles could be clearly found, which originated from the decomposition of MOF (Figure S5, Supporting Information; Figure 2b). Further examination of lattice spacings identified three characteristic values: 0.224 nm corresponding to the (111) plane of face‐centered cubic Pd,^[^ ^26^ ^]^ 0.257 nm matching the (002) plane of hexagonal close‐packed Zr, and 0.295 nm attributed to the (011) plane of tetragonal zirconia (t‐ZrO_2_).^[^ ^39^ ^]^ The t‐ZrO_2_ phase can serve as an effective support to facilitate the dispersion of Pd nanoparticles,^[^ ^40^, ^41^ ^]^ increase the specific surface area, thereby enhancing the catalytic graphitization ability and ultimately enabling precise regulation of the ultramicroporous structure. In addition, elemental analysis clearly showed that both Pd and Zr metal atoms diffused into the carbon matrix, which may be helpful to regulate the pore geometry of the CMS membrane, as well as playing a positive role in promoting gas transport as well (Figure 2c).

The XRD results of the CMS membranes showed that all CMS membranes possessed a broad peak at 21–24° (Figure 2d), corresponding to the (002) interlayer spacing of turbostratic carbon structures.^[^ ^42^, ^43^ ^]^ With the incorporation of Pd/UiO66‐COOH, the position of the 2*θ* peak displayed a positive shift, indicating that the graphite structure of the CMS formed a narrower slit‐like pore (e.g., a lower d‐spacing, 4.28 Å vs 3.69 Å). Moreover, after the carbonization, the diffraction peak associated with Pd/UiO66‐COOH emerged, denoting that Pd/UiO66‐COOH was not completely carbonized and retained some unique structural characteristics, which were consistent with the SEM results.

Raman spectroscopy was used to characterize the CMS membranes (Figure 2e; Table S4, Supporting Information), two characteristic peaks, G band at 1580 cm^−1^ and D band at 1350 cm^−1^ were observed for all the CMS membranes.^[^ ^15^ ^]^ In addition, as the content of Pd/UiO66‐COOH increased (0 – 20 wt.%), the I_G_/I_D_ ratio increased from 1.04 (PI‐550) to 1.18 (PI/Pd‐UiO66‐COOH‐20‐550), indicating that the presence of Pd/UiO‐66‐COOH in the precursor resulted in higher concentration of sp^2^‐hybridized C and smaller ultra‐micropores in the obtained MMCMS membranes.^[^ ^44^ ^]^

To investigate the detailed structure of the CMS membranes, the C 1s, N 1s, and Pd 3d peaks of the PI‐550 and PI/Pd‐UiO66‐COOH‐X‐550 MMCMS membranes were deconvoluted (Figure S6a, Supporting Information; Figure 2f),^[^ ^45^ ^]^ the C 1s spectrum was mainly deconvoluted into several peaks corresponding to sp^2^‐hybridized (284.7 eV), sp^3^‐hybridized (285.3 eV), C ‐ O (286.6 eV), and C = O (288.0 eV).^[^ ^37^ ^]^ The sp^3^/sp^2^ C ratios of the PI/Pd‐UiO66‐COOH‐X‐550 MMCMS membranes (ranging from 0.32 to 0.24) were lower than that of the pristine CMS membrane (0.46), indicating that the Pd/UiO66‐COOH induced more ordered graphitic structure,^[^ ^16^ ^]^ which was aligned with XRD and Raman analyses.

The variation in the distribution of different types of N‐species also reflected the structural fine‐tuning process of the CMS membrane upon the incorporation of Pd/UiO66‐COOH, which consequently influenced its gas transport performance.^[^ ^46^ ^]^ Therefore, the N 1s spectrum was resolved into three peaks at 398.5, 400.1, and 401.2 eV (Figure 2g), corresponding to pyridinic N, pyrrolic N, and graphitic N, respectively.^[^ ^47^ ^]^ As the content of PI/Pd‐UiO66‐COOH increased from 0 to 20 wt.%, the amounts of pyridinic N and graphitic N in the CMS membrane exhibited a volcano‐type trend. The increased amount of pyridinic N contributed to the formation of carbon defects, thereby promoting the formation of more micropores,^[^ ^48^, ^49^ ^]^ and the increase in graphitic N further suggested an enhancement in the ordering of the carbon structure. Moreover, the Pd 3d spectra confirmed the presence of Pd^0^ and Pd^2+^ in the MMCMS membranes (Figure S6b, Supporting Information), suggesting that during the carbonization process, Pd nanoparticles were partially anchored onto the carbon layers, while a fraction of Pd may likely underwent oxidized and combined with the oxygen atoms in PI to exist in the form of PdO. Notably, a negative shift in the binding energy of Pd was observed with increasing Pd/UiO66‐COOH content, leading to an increase in the electron density on its surface, which was beneficial for promoting H_2_ adsorption.^[^ ^50^ ^]^

N_2_ adsorption–desorption isotherm analysis was conducted to explore the influence of microstructure induced by Pd/UiO66‐COOH on CMS membranes (Figure 2h; Table S5, Supporting Information). The results revealed that both PI‐550 and PI/Pd‐UiO66‐COOH‐5‐550 MMCMS membranes displayed typical type I adsorption isotherms, indicative of a predominantly microporous structure with pore sizes below 2 nm. In addition, the BET surface area analysis revealed that the PI/Pd‐UiO66‐COOH‐5‐550 MMCMS membrane (744.2 m^2^ g^−1^) exhibited a reduced value compared to the pristine CMS membrane (979.0 m^2^ g^−1^), possibly due to the introduced nanofillers collapsed at high temperatures and occupied the pore spaces in the CMS membranes, causing partial physical blockage of micropores.^[^ ^51^ ^]^ Furthermore, the interaction between Pd/UiO66‐COOH and the PI chains enhanced the molecular sieving ability through optimized ultra‐micropore structure. Additionally, the pore size distribution results (Figure S7, Supporting Information) indicated that the incorporation of Pd/UiO66‐COOH significantly increased the percentage of ultra‐micropores (<7 Å), suggesting that the Langmuir structure of the MMCMS membrane became more ordered and that more ultra‐microporous structures were generated,^[^ ^33^ ^]^ which may beneficial the gas selectivity.

To further characterize the microporous structure of PI‐550 and PI/Pd‐UiO66‐COOH‐5‐550 MMCMS membranes, CO_2_ adsorption isotherms at 0 °C were evaluated as shown in Figure S8a (Supporting Information). The results demonstrated that the PI/Pd‐UiO66‐COOH‐5‐550 MMCMS membrane exhibited a higher CO_2_ adsorption capacity, which was beneficial for CO_2_ diffusion within the pores. Moreover, the pore size distribution analysis (Figure S8b, Supporting Information) revealed that the incorporation of Pd‐UiO66‐COOH effectively regulated the pore structure of the CMS membrane, specifically, the peak intensity increased within the ranges of 3–3.8 and 4–7 Å, accompanied by a reduction in average pore size.

Figure 2i presented the SAXS patterns of the PI‐550 and PI/Pd‐UiO66‐COOH‐5‐550 CMS membranes. A notable enhancement in the SAXS signal intensity was observed for the PI/Pd‐UiO66‐COOH‐5‐550 MMCMS membrane, which was ascribed to the high electron density of Pd/UiO‐66‐COOH, which altered the electron density distribution of the CMS membrane.

### 2.3 Gas Separation Performances

**Figure 3:**
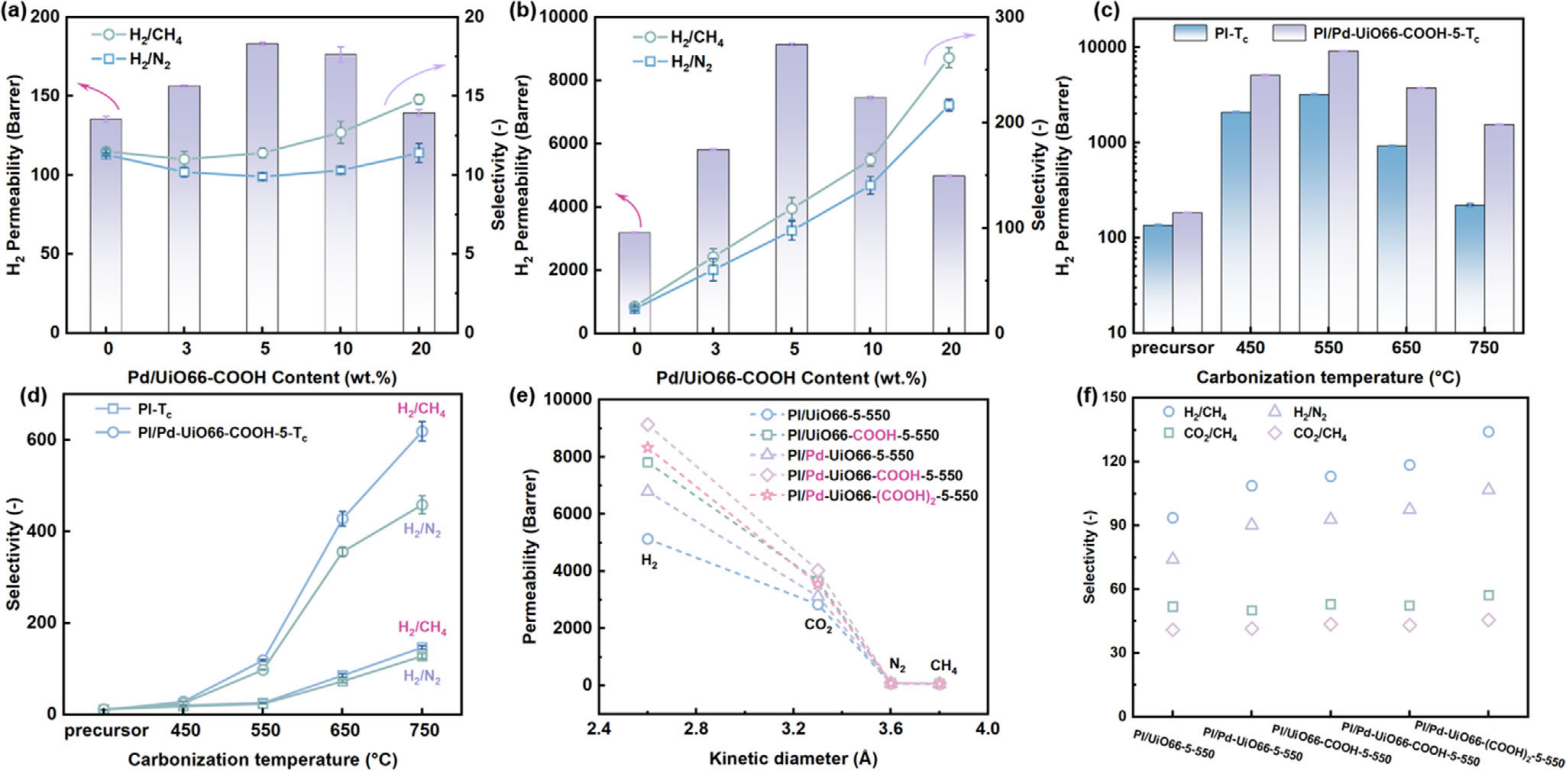
H_2_ permeability and selectivity of a) PI/Pd‐UiO66‐COOH‐X precursor membranes and b) PI/Pd‐UiO66‐COOH‐X‐550 MMCMS membranes; c) H_2_ permeability and d) selectivity of PI‐T_c_ CMS membranes and PI/Pd‐UiO66‐COOH‐5‐T_c_ MMCMS membranes; e) Gas permeability with kinetic diameter of gases, and f) H_2_/CH_4_, H_2_/N_2_, CO_2_/CH_4_ and CO_2_/N_2_ selectivity of MMCMS membranes (2 bar, 25 °C).

The effects of Pd‐UiO66‐COOH and its content on the gas separation performance of the precursor membranes and derived MMCMS membranes were examined (**Figure** **3**a,b; Table S6, Supporting Information). Similar to most MMMs, it was found that the addition of PI/Pd‐UiO66‐COOH improved the performance of the precursor membranes. The PI/Pd‐UiO66‐COOH‐5 membrane demonstrated a higher H_2_ permeability of 183.1 Barrer compared to pure PI (P_H2_ = 135.4 Barrer), this improvement could be due to the increase in fractional free volume induced by the porous Pd‐UiO66‐COOH‐5 fillers.^[^ ^52^ ^]^ Nevertheless, the excessive incorporation of Pd‐UiO66‐COOH caused its aggregation in the membrane (Figure S3, Supporting Information), which decreased the permeability of the PI/Pd‐UiO66‐COOH‐20 membrane.

In the case of MMCMS membranes, thermal treatment of the precursor membranes at 550 °C led to a notable increase in H_2_ permeability as well as H_2_/CH_4_ and H_2_/N_2_ selectivity for both pure PI and PI/Pd‐UiO66‐COOH‐X membranes. Particularly, the improvement observed in the PI/Pd‐UiO66‐COOH‐X‐550 MMCMS membranes far exceeded that of the pristine PI‐550 CMS membranes. A nearly threefold enhancement in H_2_ permeability (9134.6 Barrer vs 3191.3 Barrer), along with a significant increase in H_2_/CH_4_ and H_2_/N_2_ selectivity from 25.6 to 118.5 and from 23.4 to 97.5 was observed for the PI/Pd‐UiO66‐COOH‐5‐550 MMCMS. Likewise, as the Pd‐UiO66‐COOH content was raised from 5 to 20 wt.%, the aggregation of Pd‐UiO66‐COOH caused the obstruction of effective transport pathways, thereby decreasing the gas permeability to 4980.0 Barrer. However, it was observed that the H_2_/CH_4_ and H_2_/N_2_ selectivity were further enhanced, increasing to 261.5 and 216.7, respectively, confirming the presence of metals in the MMCMS positively promoted gas selectivity.

Figure S9 (Supporting Information) demonstrated that the presence of 5 wt.% Pd‐UiO66‐COOH in the PI matrix simultaneously improved the CO_2_ permeability as well as the CO_2_/CH_4_ and CO_2_/N_2_ selectivity of the precursor membranes and their corresponding CMS membranes. Particularly for the CMS membranes, the CO_2_ permeability increased from 2274.5 Barrer to 4033.4 Barrer, accompanied by an approximately threefold increase in CO_2_/CH_4_ and CO_2_/N_2_ selectivity. In contrast to H_2_ separation, the CO_2_ permeability of MMCMS exhibited a continuous decline with increasing the Pd‐UiO66‐COOH content from 5 to 20 wt.%, while the gas selectivity remained almost unchanged. This phenomenon might be explained by the predominant effect of Pd nanoparticles on H_2_ promotion at higher loadings, leading to a remarkable enhancement in H_2_/CH_4_ and H_2_/N_2_ selectivity. Thus, taking into account the reasonable permeability and selectivity, the mixed matrix precursor membrane with 5 wt.% Pd‐UiO66‐COOH was chosen for further investigation.

The H_2_ gas separation performance of CMS membranes fabricated from PI and PI/Pd‐UiO66‐COOH‐5 at various carbonization temperatures were evaluated, with the results illustrated in Figure 3c,d and Table S6 (Supporting Information). The CMS membranes demonstrated significantly enhanced gas permeability and selectivity relative to the precursormembranes. With elevated carbonization temperatures (450–750 °C), the H_2_ permeability exhibited a trend of initially increasing and then decreasing, indicating that the carbonization process was not yet complete at 450 °C, consistent with the findings from TGA and XRD (Figure S10, Supporting Information). The maximum H_2_ permeability of 3191.3 Barrer and 9134.6 Barrer were achieved at 550 °C for PI and PI/Pd‐UiO66‐COOH‐5 CMS membranes, respectively, establishing this temperature as the optimal carbonization condition. Concurrently, as the carbonization temperature increased from 450 to 750 °C, the selectivity of H_2_/CH_4_ and H_2_/N_2_ showed a continuous increasing trend, which was in good agreement with previously reported literature.^[^ ^29^, ^53^ ^]^ The enhanced selectivity originated from the increased densification of CMS membranes, resulting in a reduced d‐spacing between the carbon graphite layers, as evidenced by XRD results (Figure S11, Supporting Information). Compared to the PI/Pd‐UiO66‐COOH‐5 precursor membrane, the selectivity of the MMCMS membrane carbonized at 750 °C for H_2_/CH_4_ (11.9 vs 619.0) and H_2_/N_2_ (9.9 vs 458.5) increased by ≈54 and 46 times, respectively. Moreover, CO_2_ separation performance followed similar trends to H_2_ permeability and selectivity, as illustrated in Figure S10 (Supporting Information). The maximum CO_2_ permeability of 4033.4 Barrer was obtained at 550 °C, representing a tenfold enhancement over the precursor membrane.

In addition, to elucidate the dual regulatory effects of Pd‐induced Langmuir domains and ‐COOH‐mediated interfacial interactions gas separation efficiency of CMS membranes, UiO66, UiO66‐COOH, Pd/UiO66 and Pd/UiO66‐(COOH)_2_ were also incorporated as fillers into the PI matrix, resulting in the preparation of a series of MMCMS membranes. As shown in Figure 3e,f, the gas permeability of all MMCMS membranes followed the order: P_H2_ > P_CO2_ > P_N2_ > P_CH4_. This trend aligned well with the kinetic diameters of the gas molecules and the molecular sieving effect, where CH_4_ (3.8 Å) > N_2_ (3.6 Å) > CO_2_ (3.3 Å) > H_2_ (2.9 Å). Compared to the PI/UiO66‐5‐550 MMCMS membrane, the incorporation of Pd significantly enhanced the selectivity of H_2_/CH_4_ (108.7 vs 134.1) and H_2_/N_2_ (90.2 vs106.7), while the CO_2_/CH_4_ and CO_2_/N_2_ selectivity remained nearly unchanged. This phenomenon could be attributed to the synergistic effects of Pd‐induced entropy‐driven size exclusion and graphitization acceleration, which induced a more ordered structural arrangement of the carbon matrix, as further evidenced by TEM results (Figure 2b; Figures S12 and S13, Supporting Information).

Furthermore, the introduction of ‐COOH functional groups onto the nanofillers significantly enhanced the H_2_ and CO_2_ permeability. For instance, the permeability of PI/UiO66‐COOH‐5‐550 (P_H2_ = 7811.8 Barrer, P_CO2_ = 3662.1 Barrer), PI/Pd‐UiO66‐COOH‐5‐550 (P_H2_ = 9134.6 Barrer, P_CO2_ = 4033.4 Barrer), and PI/Pd‐UiO66‐(COOH)_2_‐5‐550 (P_H2_ = 8327.9 Barrer, P_CO2_ = 3551.7 Barrer) were all substantially higher than those of PI/UiO66‐5‐550 (P_H2_ = 5132.6 Barrer, P_CO2_ = 2839.5 Barrer). These results suggested that the decomposition of ‐COOH generated abundant micropores, meanwhile, the decarboxylation‐induced thermal cross‐linking effectively stabilized the carbon framework, alleviating the collapse of micropores during the carbonization process. Consequently, compared to the non‐cross‐linked PI/UiO66‐5‐550 MMCMS membrane, the PI/UiO66‐COOH‐5‐550 MMCMS membrane showed a larger d‐spacing (4.12 Å vs 4.24 Å), higher BET surface area (691.9 m^2^ g^−1^ vs 799.1 m^2^ g^−1^) and pore volume (0.29 cm^3^ g^−1^ vs 0.42 cm^3^ g^−1^), as confirmed by XRD and BET results (Figures S14 and S15 and Table S5, Supporting Information). In addition, PI/UiO66‐COOH‐5‐550 exhibited superior gas selective than PI/UiO66‐5‐550 MMCMS membrane, which could be ascribed to the thermally induced cross‐linking via decarboxylation precisely regulating the ultra‐micropore sizes, resulting in a predominant pore size distribution within the 3–5 Å range (Figure S16, Supporting Information).

**Figure 4:**
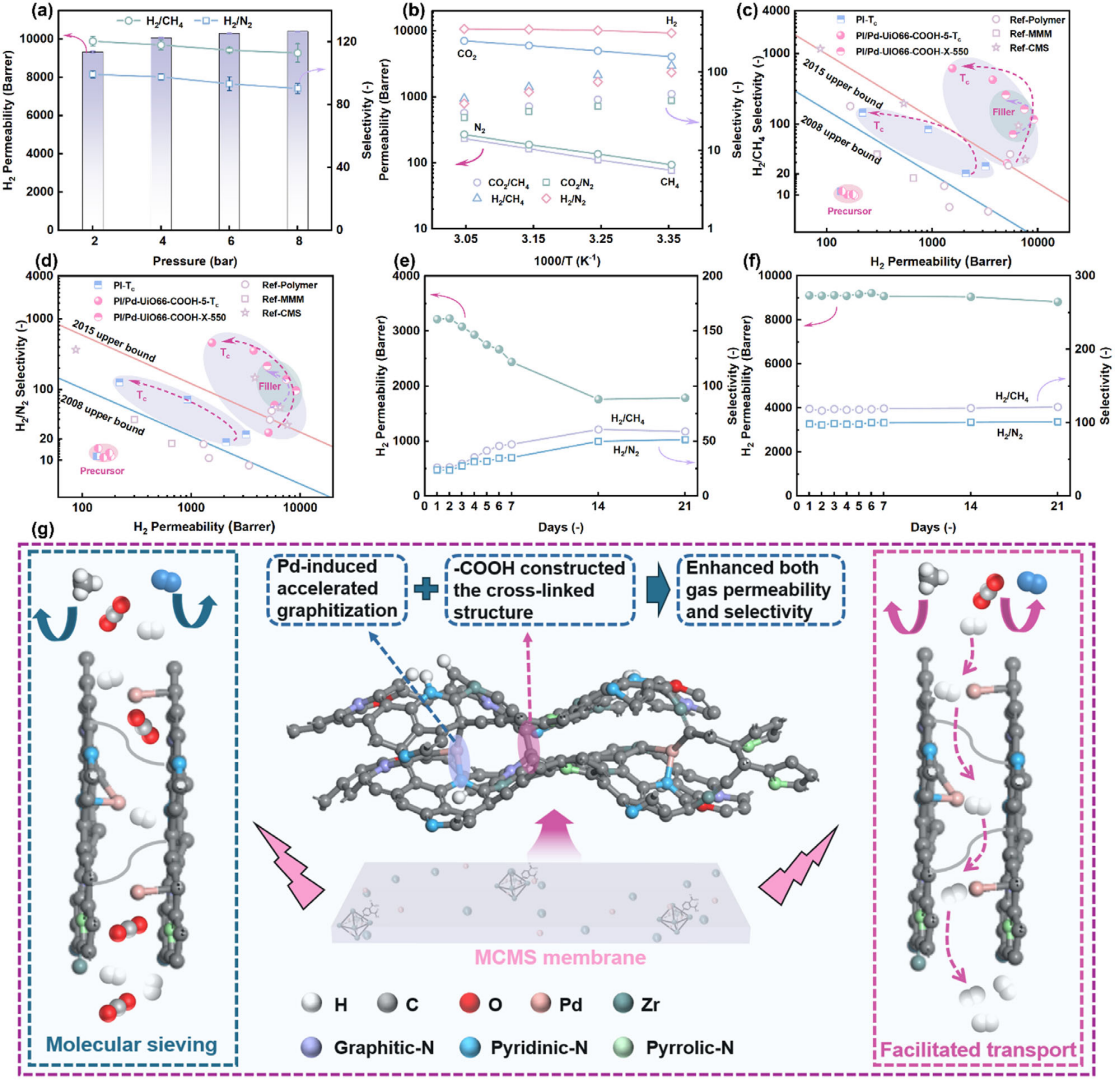
a) The effect of (a) feed pressure and b) test temperature on gas separation of PI/Pd‐UiO66‐COOH‐5‐550 MMCMS membrane; Long‐term stability of c) PI‐550 CMS membrane and d) PI/Pd‐UiO66‐COOH‐5‐550 MMCMS membrane for H_2_ separation (2 bar, 25 °C); e) H_2_/CH_4_ and f) H_2_/N_2_ separation in PI/Pd‐UiO66‐COOH‐X precursor membranes and the corresponding CMS membranes compared to the Robeson upper bound; g) The proposed gas separation mechanism of PI/Pd‐UiO66‐COOH MMCMS membrane.

Feed pressure is recognized as one of the crucial operational parameters that have an impact on the gas separation performance of membrane materials,^[^ ^54^ ^]^ thus a comprehensive investigation was carried out to explore the influence of feed pressure on the separation performance of the PI/Pd‐UiO66‐5‐550 MMCMS membrane (**Figure** **4**a; Figure S17, Supporting Information). To date, many reports have been dedicated on evaluating membrane anti‐plasticization performance within the pressure range of less than 12 bar, and exhibited that the gas separation performance remained relatively stable at 6 bar.^[^ ^12^, ^33^, ^44^, ^55^ ^]^ Thus, the gas permeation test in the current work was conducted at 2–8 bar, and the results demonstrated that elevating the feed pressure induced a slight enhancement in gas permeability (H_2_: 9322.8 to 10 399.6 Barrer, CO_2_: 4082.6 to 4423.1 Barrer), while the gas selectivity experienced a slight decrease. This trend was different from the performance changes typically observed in polymer membranes under high pressure due to the plasticization effect,^[^ ^56^, ^57^, ^58^ ^]^ suggesting that the PI/Pd‐UiO66‐5‐550 MMCMS membranes may exhibited enhanced plasticization resistance,^[^ ^33^, ^44^ ^]^ which make the MMCMS membranes promising for large‐scale industrial applications.

In addition to feed pressure, the testing temperature was also identified as a critical determinant exerting pronounced modulation effects on membrane permselectivity. As the temperature increased (25 – 55 °C), the permeability of H_2_, CO_2_, N_2_, and CH_4_ for both PI‐550 CMS and PI/Pd‐UiO66‐COOH‐5‐550 MMCMS membranes gradually increased, while the selectivity of H_2_/CH_4_, H_2_/N_2_, CO_2_/CH_4_, and CO_2_/N_2_ decreased (Figure 4b; Figure S18, Supporting Information). This was attributed to the larger transient gaps in the permeation pathways at higher temperatures.^[^ ^59^ ^]^ Notably, compared to CO_2_, the H_2_ permeability of the PI/Pd‐UiO66‐COOH‐5‐550 MMCMS membrane was less affected by the testing temperature, showing a slight increase from 9322.8 to 10 755.4 Barrer. This resulted in a significant decrease in the selectivity of H_2_/CH_4_ and H_2_/N_2_. Furthermore, the activation energy (*E*_P_) of all tested gases were calculated using the Arrhenius equation, and the results were summarized in Table S7 (Supporting Information). It was observed that for both PI‐550 and PI/Pd‐UiO66‐COOH‐5‐550 CMS membranes, the *E*_P_ values followed the order of CH_4_ > N_2_ > CO_2_ > H_2_, which was consistent with the kinetic diameters of the gases. In addition, except for a slight increase in the *E*_P_ of CO_2_, the *E_P_* of H_2_, N_2_, and CH_4_ for the PI/Pd‐UiO66‐COOH‐5‐550 MMCMS membrane (4.75, 27.95, and 33.57 kJ mol^−1^, respectively) were all lower than those of the PI‐550 CMS membrane (10.64, 34.35, and 41.99 kJ mol^−1^, respectively). This phenomenon was achieved by the decomposition of ‐COOH generated abundant micropores, and the decarboxylation‐induced thermal cross‐linking effectively mitigated the collapse of membrane pores during high‐temperature carbonization, thus preserving more effective transport pathways.^[^ ^60^ ^]^

To evaluate the performance of the prepared membranes, the H_2_‐CH_4_ and H_2_‐N_2_ separation performances of PI/Pd‐UiO66‐COOH‐X, PI/Pd‐UiO66‐COOH‐X‐550, PI‐T_c_, and PI/Pd‐UiO66‐COOH‐5‐T_c_ membranes were summarized in the Robeson upper bound plots. These results were then compared with the separation performances of reported polymer membranes, MMMs, and CMS membranes reported in the literature (Figure 4c,d; Tables S6 and S8, Supporting Information). Compared to the precursor membranes, the fabricated CMS membranes displayed a sharp increase in both gas permeability and selectivity, particularly the incorporation of Pd‐UiO66‐COOH resulted in a marked enhanced in the separation performance of the MMCMS membranes. The PI/Pd‐UiO66‐COOH‐5‐550 MMCMS membrane demonstrated a 67‐fold increase in H_2_ permeability (9134.6 vs 135.4 Barrer) compared to pure PI membranes. Simultaneously, the H_2_/CH_4_ and H_2_/N_2_ selectivity increased from 11.4 to 118.5 and from 9.9 to 97.5, respectively, surpassing the latest Robeson upper bounds. Moreover, when the carbonization temperature increased to 750 °C, the H_2_/CH_4_ and H_2_/N_2_ selectivity significantly improved to 619.0 and 458.5, respectively, while maintaining a relatively high H_2_ permeability of 1547.4 Barrer. In addition, as shown in Figure S19 and Table S9 (Supporting Information), the PI/Pd‐UiO66‐COOH‐5‐550 MMCMS membrane also demonstrated exceptional separation performance for CO_2_/CH_4_ and CO_2_/N_2_ gas pairs, surpassing the 2019 Robeson upper bounds and outperforming most reported polymer membranes, MMMs and CMS membranes. In addition, as a critical non‐renewable resource, helium (He) plays an irreplaceable role in key industrial fields such as medical technology, semiconductor manufacturing, and aerospace engineering.^[^ ^61^ ^]^ Therefore, attempts have been also made to use the PI/Pd‐UiO66‐COOH‐X‐550 MMCMS membrane to separate He, and the results exhibited that incorporating Pd/UiO66‐COOH nanofillers into the PI matrix significantly enhanced He separation performance, surpassing the latest Robeson upper bound (Figure S20, Supporting Information). The above results provide a systematic reference for the purification of natural gas, biogas, oilfield gas, coalbed methane and flue gas.

Typically, CMS membranes possessed excellent gas separation performance, however, their performance was substantially compromised by physical aging. Various molecules in the air (H_2_O and O_2_) influenced the pore structure of CMS membranes through physisorption/chemisorption, consequently leading to a decrease in permeability.^[^ ^62^, ^63^ ^]^ Therefore, stable separation performance was a critical factor to consider for long‐term applications. Figure 4e,f and Figure S21 (Supporting Information) presented the time‐dependent single gas separation performance of PI‐550 and PI/Pd‐UiO66‐COOH‐5‐550 CMS membranes. The PI‐550 CMS membrane exhibited characteristic aging behavior, as evidenced by a gradual decrease in H_2_ and CO_2_ permeability coupled with a concurrent increase in H_2_/CH_4_ (N_2_) and CO_2_/CH_4_ (N_2_) selectivity over time. In the tests conducted on the 14th day, the H_2_ and CO_2_ permeability of the membrane decreased to 1760.5 Barrer and 1087.0 Barrer, respectively, representing 55% and 48% of their initial values in fresh membranes. In addition, the permeability remained nearly unchanged during the subsequent 21‐day test, indicating that the PI‐550 CMS membranes reached a relatively stable stage by the 14th day. In contrast, the PI/Pd‐UiO66‐COOH‐5‐550 MMCMS membrane maintained stable gas permeability and selectivity at 14 days, with both H_2_ and CO_2_ permeabilities retaining over 90% of the fresh membrane even at 21st day, demonstrating excellent aging resistance. This improvement may have been due to the incorporation of Pd‐UiO66‐COOH, which increased the rigidity of the CMS membrane and prevented the collapse of the micropores, consequently slowing down the reduction in permeability and the increase in selectivity caused by the contraction of micropores and ultra‐micropores during aging.^[^ ^29^ ^]^

A series of characterization analyses were conducted to evaluate the microstructure stability of the obtained MMCMS membranes before and after aging. SEM results (Figure S22, Supporting Information) revealed no significant morphological changes in both PI‐550 CMS and PI/Pd‐UiO66‐COOH‐5‐550 MMCMS membranes after aging. However, XRD patterns (Figure S23, Supporting Information) demonstrated a notable reduction in d‐spacing for PI‐550 CMS membrane from 4.28 to 4.01 Å after aging, while the MMCMS membrane exhibited only a marginal decrease from 3.90 to 3.86 Å. These observations validated the trends observed in the long‐term stability tests and confirmed that the incorporation of Pd‐UiO66‐COOH effectively enhanced the structural stability of CMS membranes. Furthermore, as shown in Figure S24 (Supporting Information), N_2_ adsorption–desorption isotherms and pore size distribution analyses indicated a significant decrease in BET surface area along with increased ultra‐microporosity (<4 Å) for PI‐550 CMS membrane after aging, suggesting reduced permeability and enhanced selectivity. In contrast, MMCMS membranes showed minimal alterations in these parameters, a finding that corroborated well with the XRD results.

### 2.4 Gas Separation Mechanism

In addition, the probable gas separation mechanism of the PI/Pd‐UiO66‐COOH MMCMS membrane is illustrated in Figure 4g. On one hand, the Pd‐accelerated graphitization induced the rearrangement of carbon matrices into more ordered structures, which facilitated the formation of uniformly distributed sub‐nanopores. This structural evolution promoted precise molecular sieving of different gas species, thereby significantly enhancing gas selectivity. Furthermore, the decomposition of ‐COOH generated abundant micropores, menawhile the decarboxylation‐induced thermal cross‐linking stabilized the carbon framework, effectively inhibiting the collapse of micropores, thus improving both gas permeability and anti‐aging properties. On the other hand, Pd demonstrated strong adsorption and dissociation capabilities toward H_2_ molecules. As evidenced by the H_2_‐TPD profiles (Figure S25, Supporting Information), no discernible desorption peaks were observed for the PI‐550 CMS and PI/UiO66‐COOH‐5‐550 MMCMS membranes, indicating the absence of active sites capable of strong hydrogen chemisorption. In contrast, the Pd‐incorporated PI/Pd‐UiO66‐COOH‐5‐550 MMCMS membrane displayed a broad H_2_ desorption signal, which was caused by the high activation energy required for H‐Pd bond dissociation. The results indicated that H_2_ underwent dissociative chemisorption on Pd^0^ sites, forming transient H‐Pd bonds, followed by atomic hydrogen diffusion through the Pd lattice before recombining and being released on the opposite membrane side. This unique facilitated transport pathway substantially increased H_2_ permeability. Compared with conventional CMS membranes that rely on molecular sieving mechanisms,^[^ ^37^, ^64^ ^]^ the incorporation of Pd/UiO66‐COOH endowed the MMCMS membrane with enhanced H_2_ separation performance, thanks to the synergistic effects of molecular sieving and facilitated transport mechanisms.

## 3 Conclusion

In summary, novel MMCMS membranes were fabricated by incorporating Pd‐loaded COOH‐functionalized UiO‐66 (Pd/UiO‐66‐COOH) as nanofillers and were systematically characterized. It is found out that the synergistic effects of Pd‐enhanced graphitization and ‐COOH‐mediated cross‐linking collaboratively endowed the PI/Pd‐UiO66‐COOH MMCMS membrane with smaller d‐spacing, lower sp^3^/sp^2^ ratio, and reduced *E*_P_. Resulted in simultaneous enhancement in both H_2_ permeability and H_2_/CH_4_ selectivity compared to pristine PI‐derived CMS membranes. Under optimized conditions, the PI/Pd‐UiO66‐COOH‐5‐550 MMCMS membrane exhibited exceptional H_2_ permeability (9134.6 Barrer), representing a 67‐fold enhancement over the pristine PI precursor membrane, while its H_2_/CH_4_ selectivity increased from 11.5 to 118.5. Notably, long‐term stability tests revealed that the PI/Pd‐UiO66‐COOH‐5‐550 MMCMS membrane maintained stable gas separation property throughout the 14‐day test period, retaining over 90% of its original H_2_ and CO_2_ permeabilities even after 21 days, demonstrating exceptional aging resistance. The dual optimization of membrane microstructure and physical/chemical stability through nanofiller functionalization and metal loading establishes a valuable reference for novel CMS membrane material design.

## 4 Experimental Section

### Materials

1,2,4‐benzenetricarboxylic acid (99%), 1,2,4,5‐benzenetetracarboxylic acid (99%), phthalic acid (99%), zirconium tetrachloride (99%), acetic acid (99%), palladium (II) chloride (59–60%), 4,4′‐(hexafluoroisopropylidene) diphthalic anhydride (6FDA, 99%), 2,3,5,6‐tetramethyl‐p‐phenylenediamine (TMPD, 96%), m‐cresol (99%), and chloroform (≥ 99%) were supplied by Sigma–Aldrich and Macklin; Methanol (≥ 99.5%) was obtained from Tianjin Fuyu Fine Chemical Co., Ltd.; N‐methyl pyrrolidone (NMP, AR) was obtained from Chengdu Chron Chemicals Co., Ltd.; All the chemicals were used without further purification. CO_2_ (99.999%), H_2_ (99.999%), He (99.999%), N_2_ (99.99%) and CH_4_ (99.99%) used for gas permeation tests were obtained from Chengdu Xuyuan Chemical Co., ltd.

### UiO66‐COOH Synthesis

UiO66‐COOH was synthesized via a solvothermal approach adapted from established protocols.^[^ ^1^ ^]^ Typically, 1,2,4‐benzenetricarboxylic acid (3.33 mmol) and zirconium tetrachloride (3.47 mmol) were dissolved in a mixture of H_2_O (20 mL) and acetic acid (13.3 mL) in a round‐bottom flask under vigorous stirring, affording a clear solution. The resulting mixture underwent reflux condensation at 100 °C for 24 h, yielding a white crystalline precipitate. The solid product was collected by centrifugation and dispersed in methanol with continuous stirring for 72 h. Subsequently, the sample was activated by vacuum drying at 120 °C for 3 h to obtain the UiO66‐COOH. The preparation methods for UiO66‐(COOH)_2_ and UiO66 were the same as that of UiO66‐COOH, except that 1,2,4,5‐benzenetetracarboxylic acid and phthalic acid were used in place of 1,2,4‐benzenetricarboxylic acid, respectively. The prepared samples were ground into fine powder and placed in a dryer for later use.

### Pd/UiO66‐COOH Synthesis

Pd/UiO‐66‐COOH was prepared by an excessive impregnation method. A certain amount of palladium (II) chloride was dissolved in 10 mL of DI water, and then 1 g of UiO66‐COOH was added to the solution. The suspension was continuously stirred at 80 °C to allow the water to gradually evaporate. Subsequently, the remaining solid was collected and ground into fine powder, and the powder was then reduced in a H_2_ flow of 20 mL min^−1^ at 200 °C for 4 h to obtain Pd/UiO66‐COOH. The preparation processes for Pd/UiO66‐(COOH)_2_ and Pd/UiO66 were identical to that of Pd/UiO66‐COOH, except that the corresponding UiO66‐(COOH)_2_ and UiO66 were used as supports.

### Precursor Membrane Fabrication

6FDA‐TMPD PI polymer were prepared according to the procedure reported previously.^[^ ^2^ ^]^ Subsequently, 6FDA/TMPD PI polymer was added to NMP in a round‐bottom flask and stirred at room temperature for 6 h to obtain a 5 wt.% PI casting solution. A certain amount of Pd/UiO66‐COOH (3, 5, 10, 20 wt.% of Pd/UiO66‐COOH, respectively) was then added to the PI casting solution and subjected to sonicated for 1 h to form the casting solution for the preparation of MMMs. Finally, the obtained casting solution was poured into a Teflon petri dish and dried under vacuum at 60 °C for 12 h, followed by drying at 120 °C for 8 h, and 160 °C for 4 h to obtain PI/Pd/UiO66‐COOH CMS precursor membrane, and denoted as “PI/Pd‐UiO66‐COOH‐X” (X = 3, 5, 10 and 20, respectively). PI/UiO66‐COOH‐5 and PI/Pd‐UiO66‐5 membranes were prepared following the same procedure of PI/Pd‐UiO66‐COOH‐5 by using the corresponding UiO66‐COOH‐5 and Pd‐UiO66‐5 as the fillers.

### CMS Membrane Carbonization

The dried, flat PI, PI/Pd‐UiO66‐COOH‐X, PI/UiO‐66‐COOH‐5 and PI/Pd‐UiO‐66‐5 precursor membranes were cut into ≈5 cm squares respectively, and each square was sandwiched between two porous metal dishes. The assemblies were placed in a tubular furnace, which was first evacuated to remove atmospheric gases and then backfilled with N_2_ prior to increasing the temperature at a rate of 5 °C min^−1^ to the desired carbonization temperature (450, 550, 650, and 750 °C). The carbonization was maintained for 1 h, followed by quiescent cooling. During the entire carbonization process, the N_2_ flow rate was maintained at 80 mL min^−1^ to ensure the absence of O_2_. The resulting carbonized PI, PI/Pd‐UiO66‐COOH‐X, PI/UiO66‐COOH‐5 and PI/Pd‐UiO66‐5 membranes were hereafter designated as PI‐T_c_ and PI/Pd‐UiO66‐COOH‐X‐T_c_, where T_c_ denoted the carbonization temperature (expressed in °C). All CMS membranes were tested immediately to avoid the influence of physical aging.

### Membrane Characterization

The crystallographic details of the membranes were characterized using a X‐ray diffractometer (XRD, Rigaku Ultima IV, Japan) with Cu target wide‐angle diffraction (λ = 1.54 Å) operating in a 2*θ* range of 5–80°. In conjunction with the acquired scattering profiles, Bragg's law yielded the average d‐spacing (d) inside each membrane according to the formula as shown below:

(1) $$d=\frac{\lambda }{2\sin\theta }$$

where *θ* represents the angle associated with each scattering peak.

The chemical bonds of the membranes were analyzed by Fourier transform infrared (FTIR, Thermo Fisher Nicolet Is5, America), and the spectra were collected at a wavenumber range of 4000–400 cm^−1^ with an average of 32 scans at a resolution of 4 cm^−1^.

Thermal degradation profile of the membranes was characterized by thermo‐gravimetric analysis (TGA, NETZSCH‐Gerätebau GmbH, NETZSCH STA 449 F3, German), which was performed under a N_2_ environment to 800 °C at a rate of 20 °C min^−1^.

Raman spectra of CMS membranes were acquired using a laser Raman spectrometer (Thermo Fisher, DXR2xi, America) with a wavelength of 532 nm. The wavenumber range investigated was from 50–3400 cm^−1^.

The chemical compositions of the membranes were identified from X‐ray photoelectron spectroscopy (XPS, Thermo Scientific K‐Alpha, America) equipped with an Al Kα X‐ray excitation source (1486.6 eV).

The specific surface area of CMS membranes was estimated by Brunauer–Emmett–Teller (BET) model using a micromechanical ASAP2460 instrument.

The surface morphology of the membranes was performed on scanning electron microscopy (SEM, FEI Nova NanoSEM450, America) with an accelerating voltage of 15 kV. All the membrane samples were coated with gold for 60 s.

The nanostructures of the membranes were also characterized by small‐angle X‐ray scattering (SAXS, Bruker NanoSTAR U SAXS, Germany). The CMS membrane was exposed to a 14 keV beam (wavelength, λ = 0.154 nm), and the distance between the sample and detector was 2 m with a spot size of 0.5 × 0.5 mm. The azimuthal integration of the obtained 2D scattering profiles yield a 1D intensity distribution of the scattering vector (q), where q = (4π/λ)sinθ and θ is the half angle of scattering.

H_2_ temperature programmed desorption (H_2_‐TPD) was performed on tp‐5080‐B equipped with a thermal conductivity detector (TCD).

The PI‐550 CMS, PI/UiO66‐COOH‐5‐550 and PI/Pd‐UiO66‐COOH‐5‐550 MMCMS membranes were analyzed by H_2_ temperature programmed desorption (H_2_‐TPD, tp‐5080‐B, China).

The PI‐550, PI/UiO66‐5‐550, PI/Pd‐UiO66‐5‐550, and PI/Pd‐UiO66‐COOH‐5‐550 MMCMS membranes were characterized using a transmission electron microscopy (TEM, Talos F200S, America) equipped with an energy dispersive X‐ray (EDX, SUPER X, America) detector, operating at an accelerating voltage of 200 kV.

### Gas Permeation Tests

A schematic diagram of single gas permeation tests equipment was presented in Figure S1 (Supporting Information). Gas permeation experiments were performed at 25 °C and 2 bar transmembrane pressure in a constant volume/variable‐pressure apparatus. Single gas permeability can be calculated based on Equation 2:

(2) $$P_{i}=\left[{\left(\frac{dp_{d}}{\mathrm{dt}}\right)}_{t\to \infty }-{\left(\frac{dp_{d}}{\mathrm{dt}}\right)}_{\mathrm{leak}}\right]\cdot \frac{V_{d}}{A\cdot R\cdot T}\cdot \frac{l}{\left(P_{u}-P_{d}\right)}\;$$

where P_i_ is the gas permeability of component i (Barrer, 1 Barrer = 10^−10^ cm^3^ (STP) cm cm^−2^ s^−1^ cmHg^−1^), ${\left(\frac{dp_{d}}{\mathrm{dt}}\right)}_{t\to \infty }$ is the steady state rise in pressure with respect to time and ${\left(\frac{dp_{d}}{\mathrm{dt}}\right)}_{\mathrm{leak}}$ is the rate of downstream pressure increase during the leak test (cmHg s^−1^), *V*_d_ is the calibrated downstream volume (21.201 cm^3^), T is the temperature (K), R is the gas constant (0.278 cm^3^ cmHg cm^−3^ (STP) K^−1^), A is the effective membrane area (cm^2^), l is the membrane thickness (cm), *P*_u_ and *P*_d_ are the upstream and downstream pressures (cmHg), respectively.

Ideal selectivity (α^*^) is defined as the ratio of the permeability of gas penetrant i over that of j:

(3) $$\alpha^{*}=\frac{P_{i}}{P_{j}}$$

## Conflict of Interest

The authors declare no conflict of interest.

## Author Contributions

Z.D.D., X.Z.H., J.J.W., X.H.M., and W.J.J. performed conceptualization; M.D., Z.D.D., J.D.G., J.F.Z., L.Y., L.Y., and W.J.J. performed methodology; M.D., J.W., Z.K.Q., J.S., J.D.H., and J.F.Z. performed investigation; M.D. and X.H.M. performed visualization; Z.D.D., X.Z.H., J.J.W., and J.F.Z. performed supervision; M.D. wrote the original draft; Z.D.D. reviewed and edited the final manuscript.

## Supporting information



Supporting Information

## Data Availability

The data that support the findings of this study are available from the corresponding author upon reasonable request.
