# Supplementary material for: Cascade Promotion of Gas Separation Performances in CMS Membranes: MOFs With Functional Groups and Loaded Noble Metals
Source: Adv Sci (Weinh). 2025 Jun 5;12(32):e03471. doi: 10.1002/advs.202503471 (PMC12407267; doi:10.1002/advs.202503471)
Supplement: Supplementary file 1 — Supporting Information [file ADVS-12-e03471-s001.docx]

**Supplementary Information**

**Cascade Promotion of Gas Separation Performances in CMS Membranes: MOFs with Functional Groups and Loaded Noble Metals**

*Min Deng, Jing Wei, Jundong Guo, Zikang Qin, Jia Song, Junfeng Zheng, Lin Yang, Lu Yao, Wenju Jiang, Xiaohua Ma, Xuezhong He,* Jiadai He, Jianjian Wang,* Zhongde Dai**

Min Deng, Jing Wei

College of Architecture and Environment, Sichuan University, Chengdu 610065, China

National Engineering Research Centre for Flue Gas Desulfurization, Chengdu 610065, China

Carbon Neutral Technology Innovation Center of Sichuan, Chengdu 610065, China

College of Carbon Neutrality Future Technology, Sichuan University, Chengdu 610065, China

Jundong Guo

DongFang Boiler Co., Ltd., Zigong, Sichuan, 643001, China

Zikang Qin, Jia Song

National Engineering Research Centre for Flue Gas Desulfurization, Chengdu 610065, China

Carbon Neutral Technology Innovation Center of Sichuan, Chengdu 610065, China

College of Carbon Neutrality Future Technology, Sichuan University, Chengdu 610065, China

Junfeng Zheng, Lin Yang, Lu Yao, Wenju Jiang,

National Engineering Research Centre for Flue Gas Desulfurization, Chengdu 610065, China

Carbon Neutral Technology Innovation Center of Sichuan, Chengdu 610065, China

College of Carbon Neutrality Future Technology, Sichuan University, Chengdu 610065, China

Xiaohua Ma

State Key Laboratory of Separation Membranes and Membrane Processes, School of Materials Science and Engineering, Tiangong University, Tianjin 300387, PR China

Xuezhong He^*^

Department of Chemical Engineering, Guangdong Technion-Israel Institute of Technology, 241 Daxue Road, Shantou, Guangdong 515063, China

Jiadai He, Jianjian Wang^*^

School of Chemistry and Chemical Engineering, Chongqing University, Chongqing 401331, China

Zhongde Dai^*^

National Engineering Research Centre for Flue Gas Desulfurization, Chengdu 610065, China

Carbon Neutral Technology Innovation Center of Sichuan, Chengdu 610065, China

College of Carbon Neutrality Future Technology, Sichuan University, Chengdu 610065, China

*Corresponding Author:

Xuezhong He: [xuezhong.he@gtiit.edu.cn](mailto:xuezhong.he@gtiit.edu.cn)

Jianjian Wang: wangjianjian@cqu.edu.cn

Zhongde Dai: [zhongde.dai@scu.edu.cn](mailto:zhongde.dai@scu.edu.cn)

**Table of contents**

[1. Materials 4](#_Toc199401852)

[2. UiO66-COOH synthesis 4](#_Toc199401853)

[3. Pd/UiO66-COOH synthesis 5](#_Toc199401854)

[4. Precursor membrane fabrication 5](#_Toc199401855)

[5. CMS membrane carbonization 5](#_Toc199401856)

[6. Membrane characterization 6](#_Toc199401857)

[7. Gas permeation tests 7](#_Toc199401858)

[Figure S1. Schematic diagram of single gas permeation test equipment.^[3]^ 9](#_Toc199401859)

[Figure S2. SEM surface images of PI/Pd-UiO66-COOH-X precursor membranes. 10](#_Toc199401860)

[Figure S3. SEM surface images (insert: cross-section image) of PI/Pd-UiO66-COOH-X-550 MMCMS membranes. 11](#_Toc199401861)

[Figure S4. EDS mapping of PI/Pd-UiO66-COOH-X-550 MMCMS membranes. 12](#_Toc199401862)

[Figure S5. (a) SEM image of Pd/UiO66-COOH; (b) TEM images of PI/Pd-UiO66-COOH-5-550 MMCMS membrane. 13](#_Toc199401863)

[Figure S6. (a) XPS survey spectra, (b) High-resolution Pd 3d XPS spectra of PI/Pd-UiO66-COOH-X-550 MMCMS membranes. 14](#_Toc199401864)

[Figure S7. Pore size distribution curve of PI-550 and PI/Pd-UiO66-COOH-5-550 CMS membranes. 15](#_Toc199401865)

[Figure S8. (a) CO_2_ sorption isotherms measured at 0 °C and (b) pore size distribution of PI-550 CMS and PI/Pd-UiO66-COOH-5-550 MMCMS membranes. 16](#_Toc199401866)

[Figure S9. CO_2_ permeability and selectivity of (a) PI/Pd-UiO66-COOH-X precursor membranes, (b) PI/Pd-UiO66-COOH-X-550 MMCMS membranes. 17](#_Toc199401867)

[Figure S10. (a) CO_2_ permeability, and (b) CO_2_/CH_4_, CO_2_/N_2_ selectivity of PI-T_c_ CMS membranes and PI/Pd-UiO66-COOH-5-T_c_ MMCMS membranes obtained under different carbonization temperatures. 18](#_Toc199401868)

[Figure S11. XRD pattern of (a) PI-T_c_ and (b) PI/Pd-UiO66-COOH-5-T_c_ MMCMS membranes obtained under different carbonization temperatures. 19](#_Toc199401869)

[Figure S12. (a) SEM image of UiO-66; (b) TEM and (c) HRTEM images of PI/UiO66-5-550 MMCMS membrane; (d) HAADF-STEM image of PI/UiO66-5-550 MMCMS membrane and corresponding EDS mappings. 20](#_Toc199401870)

[Figure S13. (a) SEM image of Pd/UiO-66; (b) TEM and (c) HRTEM images of PI/Pd-UiO66-5-550 MMCMS membrane; (d) HAADF-STEM image of PI/Pd-UiO66-5-550 membrane and corresponding EDS mappings. 21](#_Toc199401871)

[Figure S14. XRD patterns of PI/UiO66-5-550, PI/UiO66-COOH-5-550, PI/Pd-UiO66-5-550 and PI/Pd-UiO66-COOH-5-550 MMCMS membranes. 22](#_Toc199401872)

[Figure S15. N_2_ adsorption/desorption isotherms of PI/UiO66-5-550, PI/UiO66-COOH-5-550, and PI/Pd-UiO66-COOH-5-550 MMCMS membranes. 23](#_Toc199401873)

[Figure S16. (a) CO_2_ sorption isotherms measured at 0 °C and (b) pore size distribution of PI/UiO66-5-550, PI/UiO66-COOH-5-550 and PI/Pd-UiO66-COOH-5-550 MMCMS membranes. 24](#_Toc199401874)

[Figure S17. The effect of feed pressure on CO_2_ separation of PI/Pd-UiO66-COOH-5-550 MMCMS membrane (gas permeation test carried out at 25 °C). 25](#_Toc199401875)

[Figure S18. The effect of test temperature on gas separation of PI-550 CMS membrane (tested at a feed pressure of 2 bar). 26](#_Toc199401876)

[Figure S19. (a) CO_2_/CH_4_ and (b) CO_2_/N_2_ separation performances of PI/Pd-UiO66-COOH-X precursor membranes and their derived MMCMS membranes compared to the Robeson upper bound. 27](#_Toc199401877)

[Figure S20. (a) He permeability and selectivity of PI/Pd-UiO66-COOH-X-550 MMCMS membranes; (b) He/CH_4_ and (c) He/N_2_ separation performances of PI/Pd-UiO66-COOH-X-550 MMCMS membranes compared to the Robeson upper bound. 28](#_Toc199401878)

[Figure S21. Long-term stability of (a)PI-550 CMS membrane and (b) PI/Pd-UiO66-COOH-5-550 MMCMS membrane for CO_2_ separation (tested at a feed pressure of 2 bar at 25 °C). 29](#_Toc199401879)

[Figure S22. SEM surface images of PI-550 CMS and PI/Pd-UiO66-COOH-X-550 MMCMS membranes before and after aging. 30](#_Toc199401880)

[Figure S23. XRD patterns of PI-550 CMS and PI/Pd-UiO66-COOH-X-550 MMCMS membranes before and after aging. 31](#_Toc199401881)

[Figure S24. (a) N_2_ adsorption/desorption isotherms and (b) pore size distribution curves of PI-550 CMS and PI/Pd-UiO66-COOH-X-550 MMCMS membranes before and after aging. 32](#_Toc199401882)

[Figure S25. H2-TPD profiles of PI-550 CMS, PI/UiO66-COOH-5-550 and PI/Pd-UiO66-COOH-5-550 MMCMS membranes. 33](#_Toc199401883)

[Table S1 Metal loadings (ICP) of various samples. 34](#_Toc199401884)

[Table S2 d-spacing of various precursor membranes from XRD patterns. 35](#_Toc199401885)

[Table S3. Peak assignment in the FTIR results. 36](#_Toc199401886)

[Table S4 I_G_/I_D_ values in the Raman results from pristine CMS and MMCMS membranes. 37](#_Toc199401887)

[Table S5 Textural properties of various materials. 38](#_Toc199401888)

[Table S6 Single-gas separation performances of this work. 39](#_Toc199401889)

[Table S7 Activation energy of permeation for different gases in PI-550 CMS membrane and PI/Pd-UiO66-COOH-5-550 MMCMS membranes. 40](#_Toc199401890)

[Table S8 Comparison of H_2_ separation performance of polymer membranes, MMMs and CMS membranes derived from different precursors. 41](#_Toc199401891)

[Table S9 Comparison of CO_2_ separation performance of polymer membranes, MMMs and CMS membranes derived from different precursors. 42](#_Toc199401892)

[Reference 43](#_Toc199401893)

# 1. Materials

1,2,4-benzenetricarboxylic acid (99%), 1,2,4,5-benzenetetracarboxylic acid (99%), phthalic acid (99%), zirconium tetrachloride (99%), acetic acid (99%), palladium (II) chloride (59-60%), 4,4’-(hexafluoroisopropylidene) diphthalic anhydride (6FDA, 99%), 2,3,5,6-tetramethyl-p-phenylenediamine (TMPD, 96%), m-cresol (99%), and chloroform (≥ 99%) were supplied by Sigma-Aldrich and Macklin; Methanol (≥ 99.5%) was obtained from Tianjin Fuyu Fine Chemical Co., Ltd.; N-methyl pyrrolidone (NMP, AR) was obtained from Chengdu Chron Chemicals Co., Ltd.; All the chemicals were used without further purification. CO_2_ (99.999%), H_2_ (99.999%), He (99.999%), N_2_ (99.99%) and CH_4_ (99.99%) used for gas permeation tests were obtained from Chengdu Xuyuan Chemical Co., ltd.

# 2. UiO66-COOH synthesis

UiO66-COOH was synthesized via a solvothermal approach adapted from established protocols.^[1]^ Typically, 1,2,4-benzenetricarboxylic acid (3.33 mmol) and zirconium tetrachloride (3.47 mmol) were dissolved in a mixture of H_2_O (20 mL) and acetic acid (13.3 mL) in a round-bottom flask under vigorous stirring, affording a clear solution. The resulting mixture underwent reflux condensation at 100 °C for 24 h, yielding a white crystalline precipitate. The solid product was collected by centrifugation and dispersed in methanol with continuous stirring for 72 h. Subsequently, the sample was activated by vacuum drying at 120 °C for 3 h to obtain the UiO66-COOH. The preparation methods for UiO66-(COOH)_2_ and UiO66 were the same as that of UiO66-COOH, except that 1,2,4,5-benzenetetracarboxylic acid and phthalic acid were used in place of 1,2,4-benzenetricarboxylic acid, respectively. The prepared samples were ground into fine powder and placed in a dryer for later use.

# 3. Pd/UiO66-COOH synthesis

Pd/UiO-66-COOH was prepared by an excessive impregnation method. A certain amount of palladium (II) chloride was dissolved in 10 mL of DI water, and then 1 g of UiO66-COOH was added to the solution. The suspension was continuously stirred at 80 °C to allow the water to gradually evaporate. Subsequently, the remaining solid was collected and ground into fine powder, and the powder was then reduced in a H_2_ flow of 20 mL/min at 200 °C for 4 h to obtain Pd/UiO66-COOH. The preparation processes for Pd/UiO66-(COOH)_2_ and Pd/UiO66 were identical to that of Pd/UiO66-COOH, except that the corresponding UiO66-(COOH)_2_ and UiO66 were used as supports.

# 4. Precursor membrane fabrication

6FDA-TMPD PI polymer were prepared according to the procedure reported previously ^[2]^. Subsequently, 6FDA/TMPD PI polymer was added to NMP in a round-bottom flask and stirred at room temperature for 6 h to obtain a 5 wt.% PI casting solution. A certain amount of Pd/UiO66-COOH (3, 5, 10, 20 wt.% of Pd/UiO66-COOH respectively) was then added to the PI casting solution and subjected to sonicated for 1 h to form the casting solution for the preparation of MMMs. Finally, the obtained casting solution was poured into a Teflon petri dish and dried under vacuum at 60 °C for 12 h, followed by drying at 120 °C for 8 h, and 160 °C for 4 h to obtain PI/Pd/UiO66-COOH CMS precursor membrane, and denoted as “PI/Pd-UiO66-COOH-X” (X= 3, 5, 10 and 20, respectively). PI/UiO66-COOH-5 and PI/Pd-UiO66-5 membranes were prepared following the same procedure of PI/Pd-UiO66-COOH-5 by using the corresponding UiO66-COOH-5 and Pd-UiO66-5 as the fillers.

# 5. CMS membrane carbonization

The dried, flat PI, PI/Pd-UiO66-COOH-X, PI/UiO-66-COOH-5 and PI/Pd-UiO-66-5 precursor membranes were cut into ~5 cm squares respectively, and each square was sandwiched between two porous metal dishes. The assemblies were placed in a tubular furnace, which was first evacuated to remove atmospheric gases and then backfilled with N_2_ prior to increasing the temperature at a rate of 5 °C/min to the desired carbonization temperature (450, 550, 650, and 750 °C). The carbonization was maintained for 1 h, followed by quiescent cooling. During the entire carbonization process, the N_2_ flow rate was maintained at 80 mL/min to ensure the absence of O_2_. The resulting carbonized PI, PI/Pd-UiO66-COOH-X, PI/UiO66-COOH-5 and PI/Pd-UiO66-5 membranes were hereafter designated as PI-T_c_ and PI/Pd-UiO66-COOH-X-T_c_, where T_c_ denoted the carbonization temperature (expressed in °C). All CMS membranes were tested immediately to avoid the influence of physical aging.

# 6. Membrane characterization

The crystallographic details of the membranes were characterized using a X-ray diffractometer (XRD, Rigaku Ultima IV, Japan) with Cu target wide-angle diffraction (λ=1.54 Å) operating in a 2θ range of 5-80°. In conjunction with the acquired scattering profiles, Bragg’s law yielded the average d-spacing (d) inside each membrane according to the formula as shown below:

$$\text{d=}\frac{\text{λ}}{\text{2sin} \text{θ}}\text{ }\text{ (}\text{1)}$$

where θ represents the angle associated with each scattering peak.

The chemical bonds of the membranes were analyzed by Fourier transform infrared (FTIR, Thermo Fisher Nicolet Is5, America), and the spectra were collected at a wavenumber range of 4000-400 cm^-1^ with an average of 32 scans at a resolution of 4 cm^-1^.

Thermal degradation profile of the membranes was characterized by thermo-gravimetric analysis (TGA, NETZSCH-Gerätebau GmbH, NETZSCH STA 449 F3, German), which was performed under a N_2_ environment to 800 °C at a rate of 20 °C min^-1^.

Raman spectra of CMS membranes were acquired using a laser Raman spectrometer (Thermo Fisher, DXR2xi, America) with a wavelength of 532 nm. The wavenumber range investigated was from 50 ~ 3400 cm^-1^.

The chemical compositions of the membranes were identified from X-ray photoelectron spectroscopy (XPS, Thermo Scientific K-Alpha, America) equipped with an Al Kα X-ray excitation source (1486.6 eV).

The specific surface area of CMS membranes was estimated by Brunauer-Emmett-Teller (BET) model using a micromechanical ASAP2460 instrument.

The surface morphology of the membranes was performed on scanning electron microscopy (SEM, FEI Nova NanoSEM450, America) with an accelerating voltage of 15 kV. All the membrane samples were coated with gold for 60 seconds.

The nanostructures of the membranes were also characterized by small-angle X-ray scattering (SAXS, Bruker NanoSTAR U SAXS, Germany). The CMS membrane was exposed to a 14 keV beam (wavelength, λ = 0.154 nm), and the distance between the sample and detector was 2 m with a spot size of 0.5 × 0.5 mm. The azimuthal integration of the obtained 2D scattering profiles yield a 1D intensity distribution of the scattering vector (q), where q = (4π/λ)sinθ and θ is the half angle of scattering.

H_2_ temperature programmed desorption (H_2_-TPD) was performed on tp-5080-B equipped with a thermal conductivity detector (TCD).

The PI-550 CMS, PI/UiO66-COOH-5-550 and PI/Pd-UiO66-COOH-5-550 MMCMS membranes were analyzed by H_2_ temperature programmed desorption (H_2_-TPD, tp-5080-B, China).

The PI-550, PI/UiO66-5-550, PI/Pd-UiO66-5-550, and PI/Pd-UiO66-COOH-5-550 MMCMS membranes were characterized using a transmission electron microscopy (TEM, Talos F200S, America) equipped with an energy dispersive X-ray (EDX, SUPER X, America) detector, operating at an accelerating voltage of 200 kV.

# 7. Gas permeation tests

A schematic diagram of single gas permeation tests equipment was presented in **Figure S1**. Gas permeation experiments were performed at 25 °C and 2 bar transmembrane pressure in a constant volume/variable-pressure apparatus. Single gas permeability can be calculated based on equation (2):

$$\text{P}_{\text{i}}\text{=}\left[ \left( \frac{\text{dp}_{\text{d}}}{\text{dt}} \right)_{\text{t→∞}}\text{-}\left( \frac{\text{dp}_{\text{d}}}{\text{dt}} \right)_{\text{leak}} \right]\text{·}\frac{\text{V}_{\text{d}}}{\text{A·R·T}}\text{·}\frac{\text{l}}{\text{(}\text{P}_{\text{u}}\text{-}\text{P}_{\text{d}}\text{)}}\text{ }\left( \text{2} \right)$$

where P_i_ is the gas permeability of component i (Barrer, 1 Barrer = 10^-10^ cm^3^ (STP) cm cm^-2^ s^-1^ cmHg^-1^), $\left( \frac{\text{dp}_{\text{d}}}{\text{dt}} \right)_{\text{t→∞}}$ is the steady state rise in pressure with respect to time and $\left( \frac{\text{dp}_{\text{d}}}{\text{dt}} \right)_{\text{leak}}$ is the rate of downstream pressure increase during the leak test (cmHg s^-1^), V_d_ is the calibrated downstream volume (21.201 cm^3^), T is the temperature (K), R is the gas constant (0.278 cm^3^ cmHg cm^-3^ (STP) K^-1^), A is the effective membrane area (cm^2^), l is the membrane thickness (cm), P_u_ and P_d_ are the upstream and downstream pressures (cmHg), respectively.

Ideal selectivity (α^*^) is defined as the ratio of the permeability of gas penetrant i over that of j:

$$\text{α}^{\text{*}}\text{=}\frac{\text{P}_{\text{i}}}{\text{P}_{\text{j}}}\text{ }\left( \text{3} \right)$$

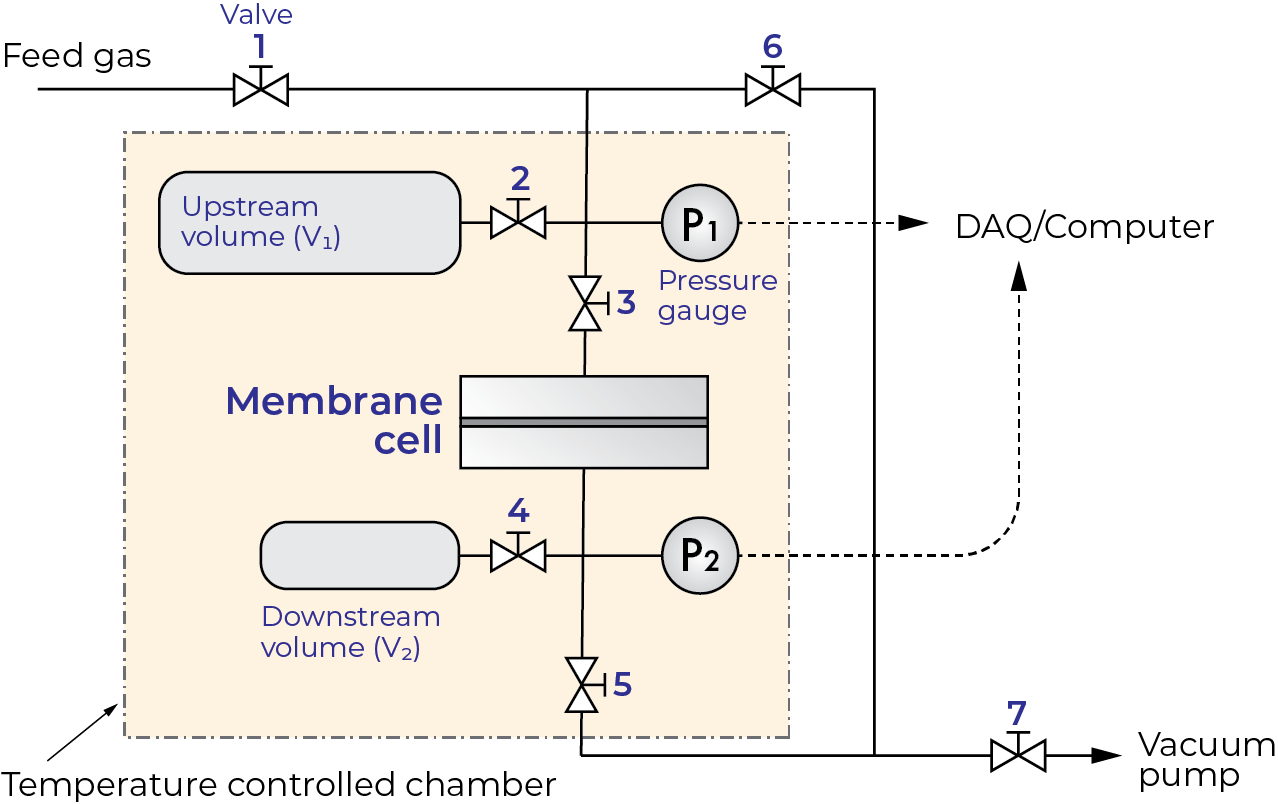


# Figure S1. Schematic diagram of single gas permeation test equipment.^[3]^


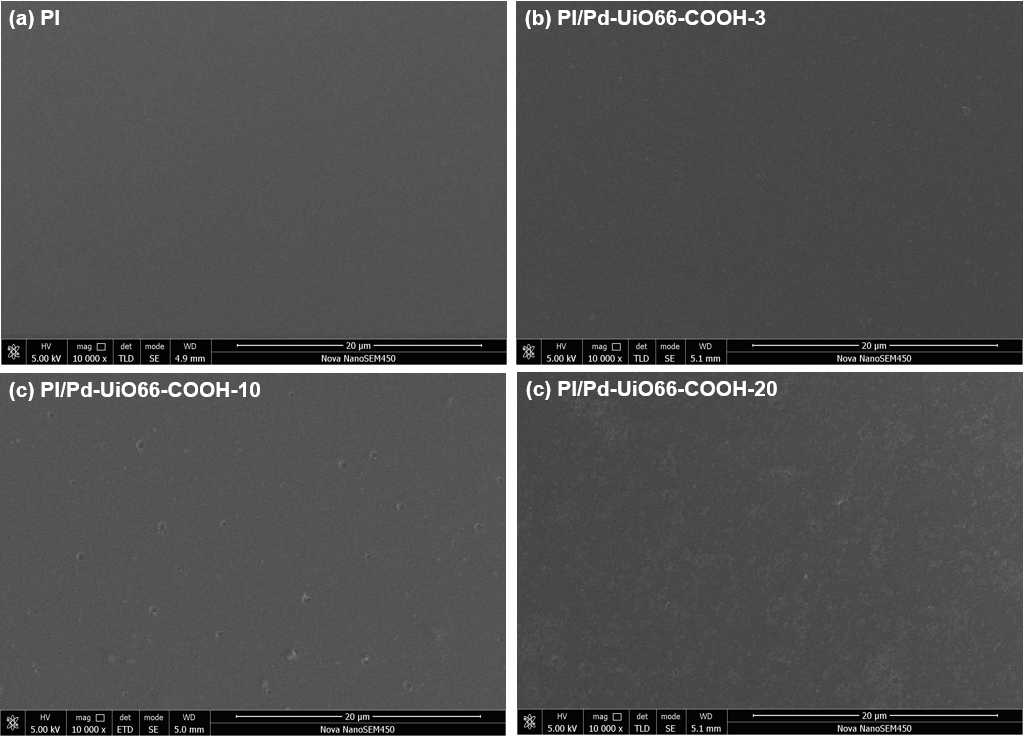


# Figure S2. SEM surface images of PI/Pd-UiO66-COOH-X precursor membranes.


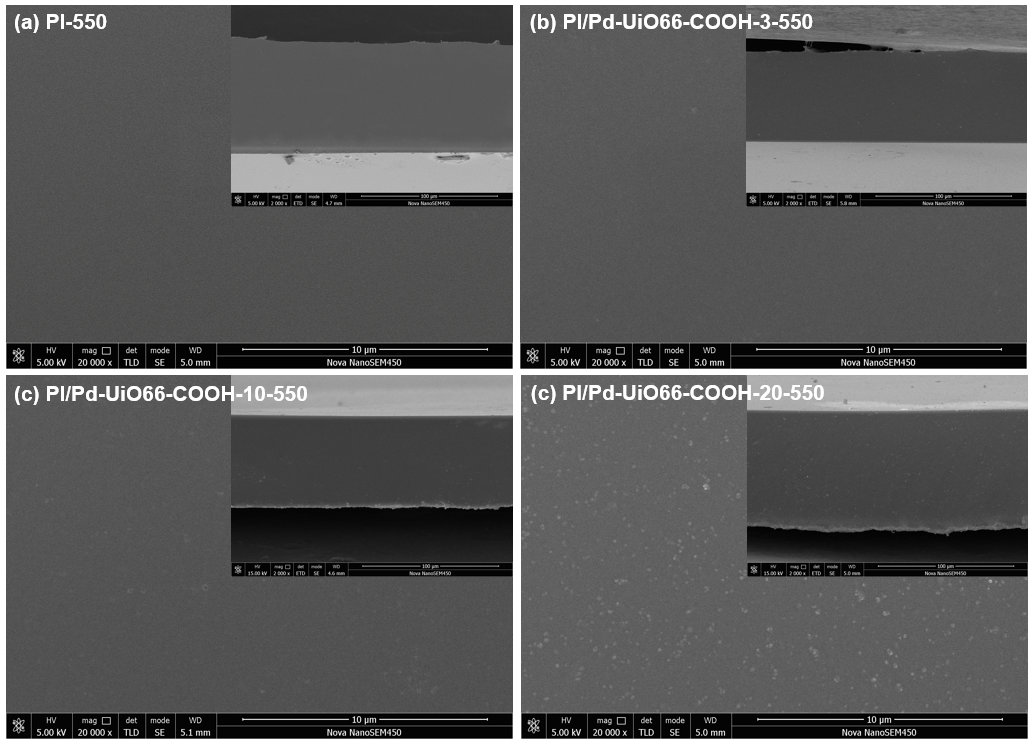


# Figure S3. SEM surface images (insert: cross-section image) of PI/Pd-UiO66-COOH-X-550 MMCMS membranes.


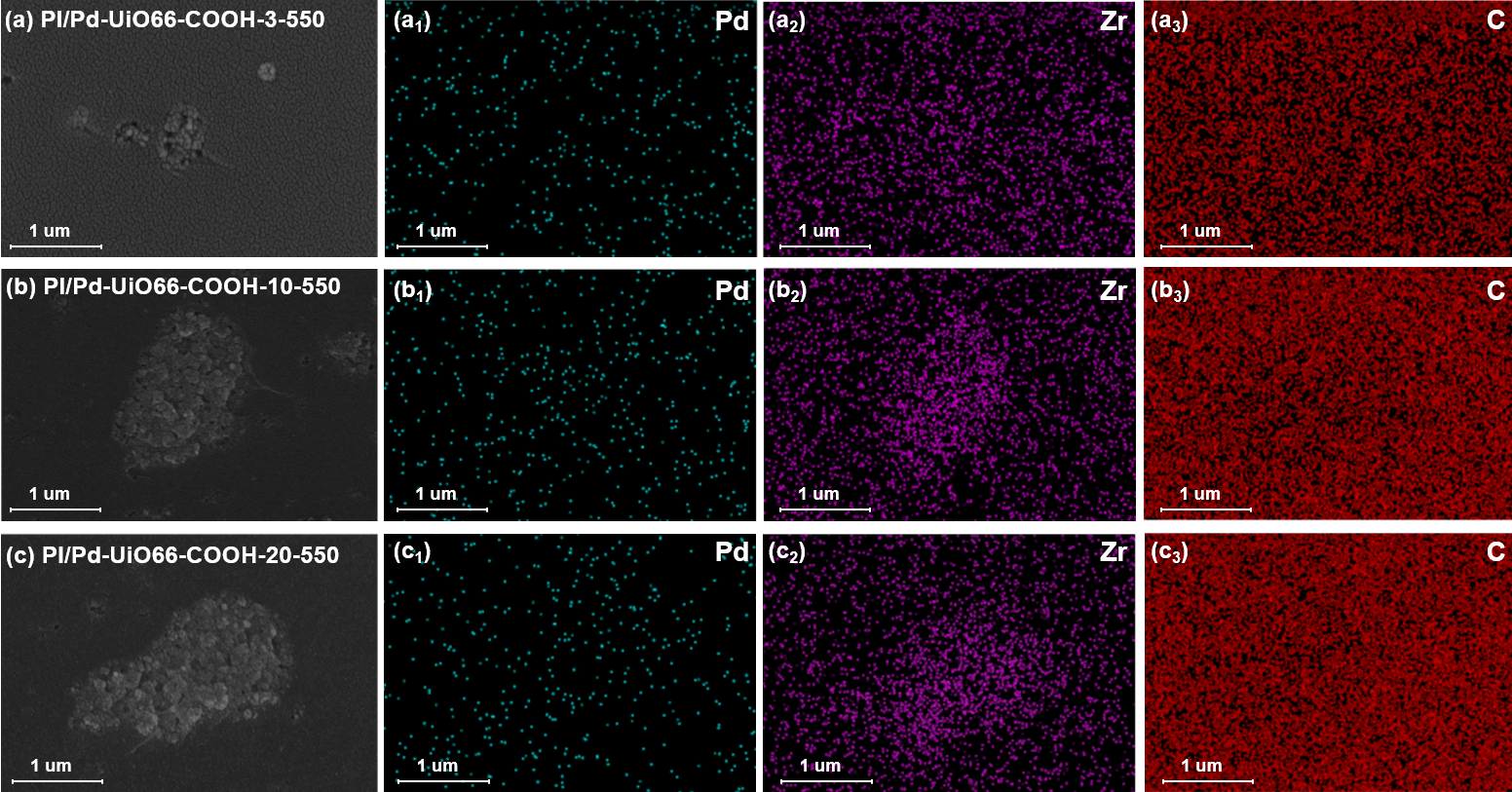


# Figure S4. EDS mapping of PI/Pd-UiO66-COOH-X-550 MMCMS membranes.


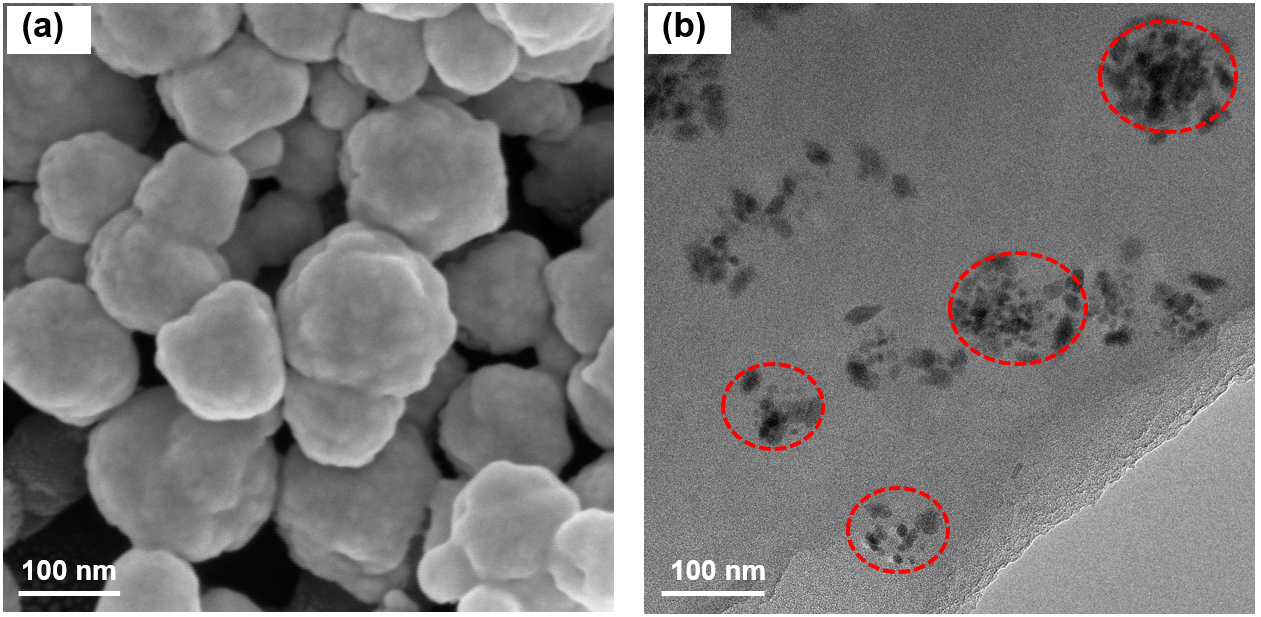


# Figure S5. (a) SEM image of Pd/UiO66-COOH; (b) TEM images of PI/Pd-UiO66-COOH-5-550 MMCMS membrane.


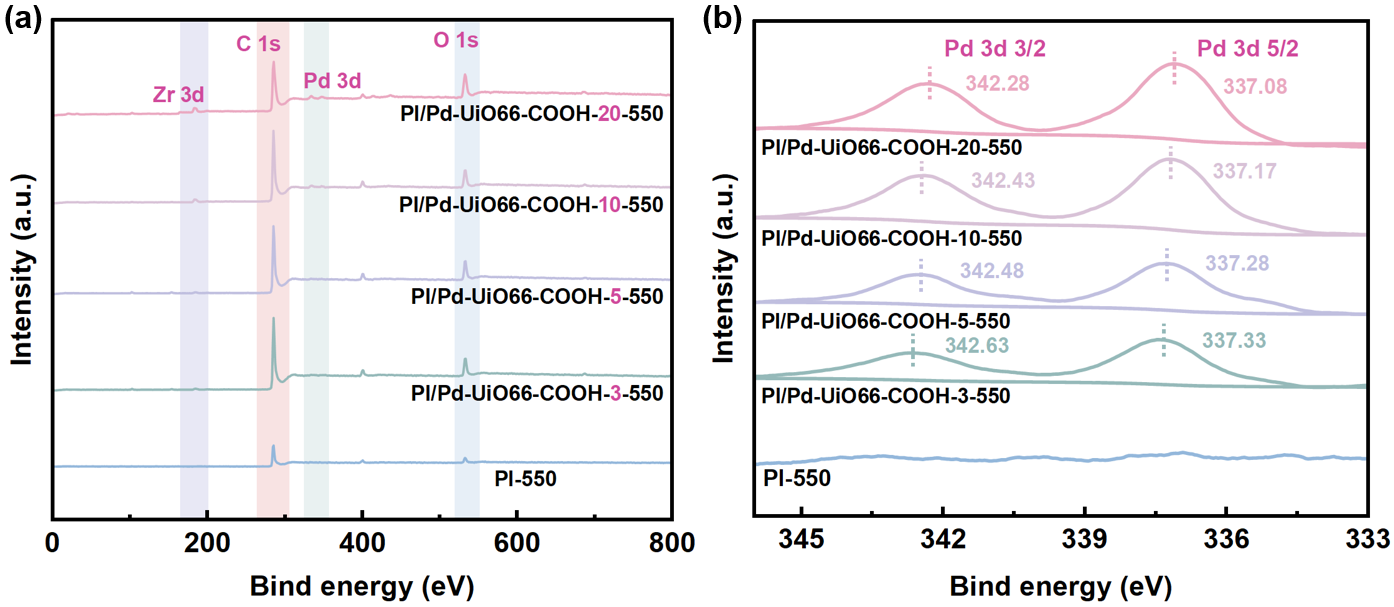


# Figure S6. (a) XPS survey spectra, (b) High-resolution Pd 3d XPS spectra of PI/Pd-UiO66-COOH-X-550 MMCMS membranes.


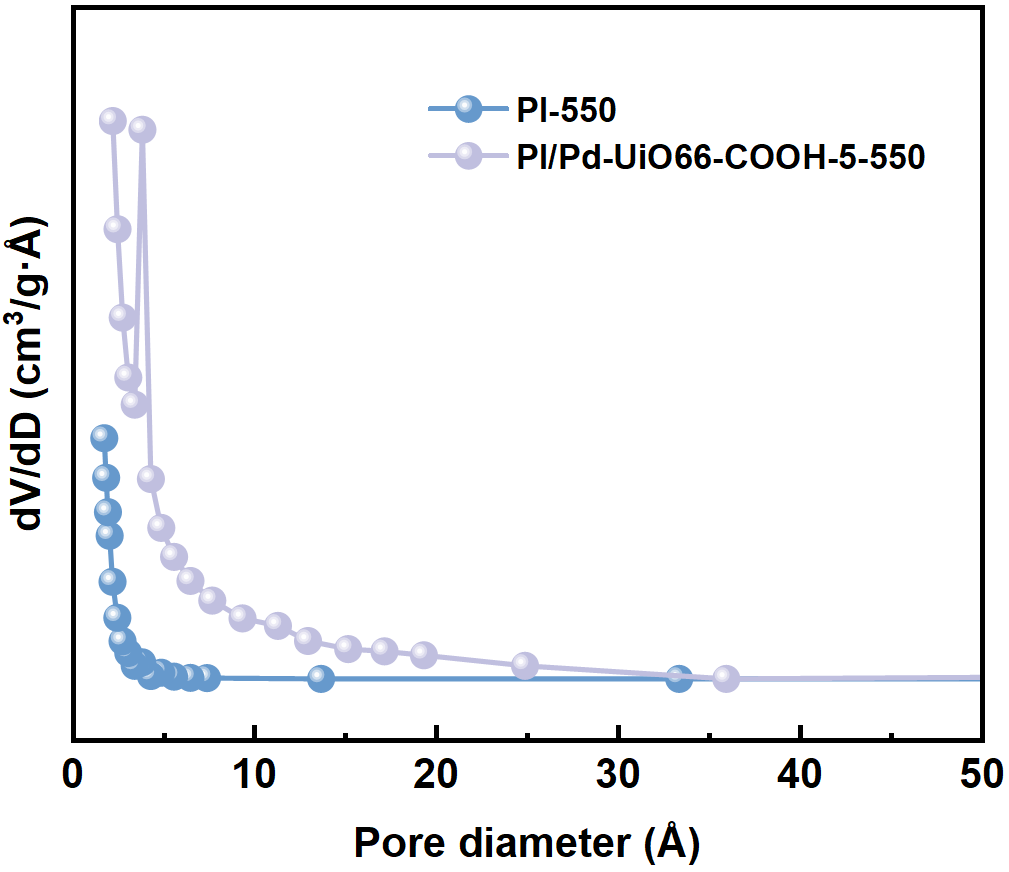


# Figure S7. Pore size distribution curve of PI-550 and PI/Pd-UiO66-COOH-5-550 CMS membranes.


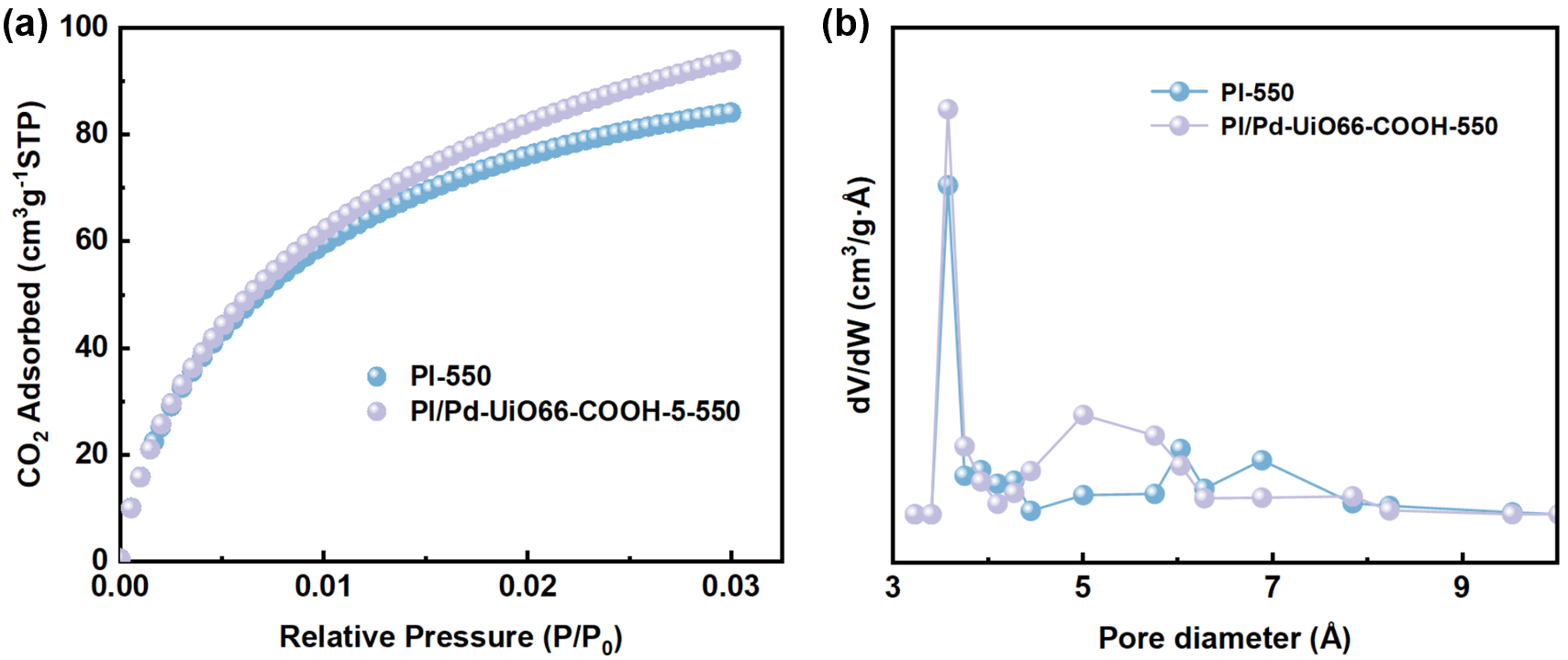


# Figure S8. (a) CO_2_ sorption isotherms measured at 0 °C and (b) pore size distribution of PI-550 CMS and PI/Pd-UiO66-COOH-5-550 MMCMS membranes.


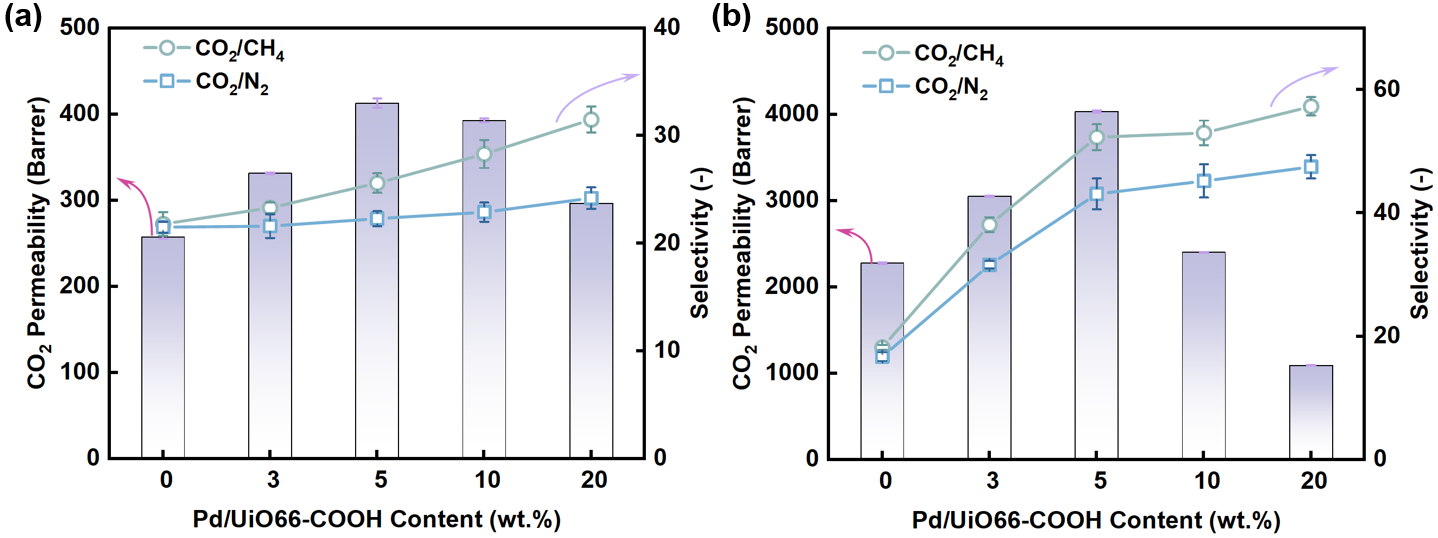


# Figure S9. CO_2_ permeability and selectivity of (a) PI/Pd-UiO66-COOH-X precursor membranes, (b) PI/Pd-UiO66-COOH-X-550 MMCMS membranes.


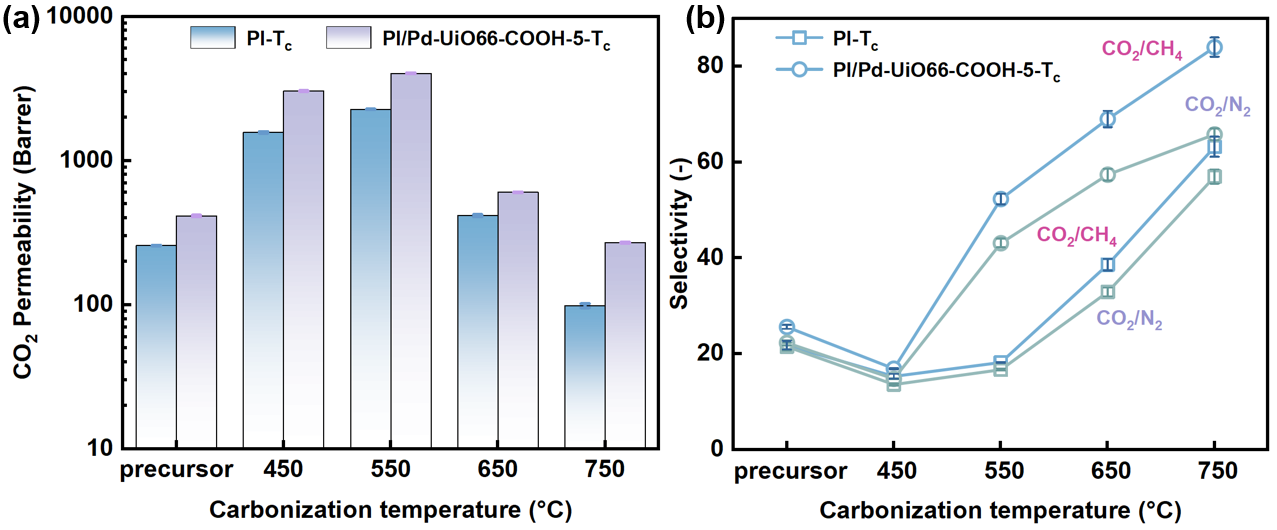


# Figure S10. (a) CO_2_ permeability, and (b) CO_2_/CH_4_, CO_2_/N_2_ selectivity of PI-T_c_ CMS membranes and PI/Pd-UiO66-COOH-5-T_c_ MMCMS membranes obtained under different carbonization temperatures.


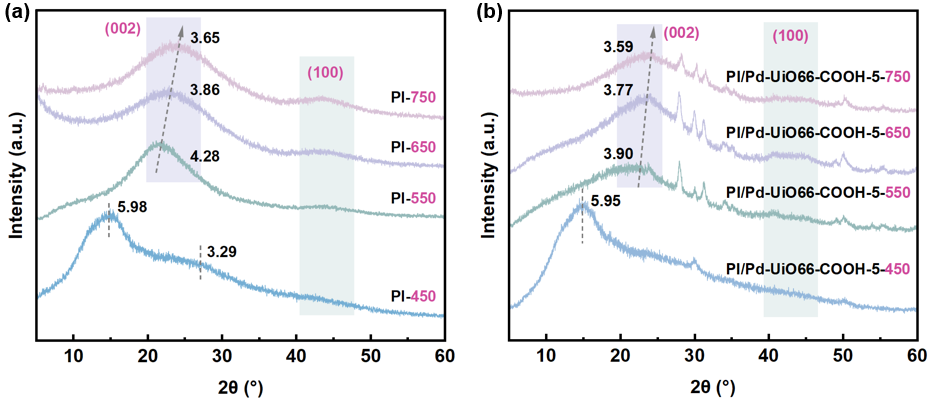


# Figure S11. XRD pattern of (a) PI-T_c_ and (b) PI/Pd-UiO66-COOH-5-T_c_ MMCMS membranes obtained under different carbonization temperatures.


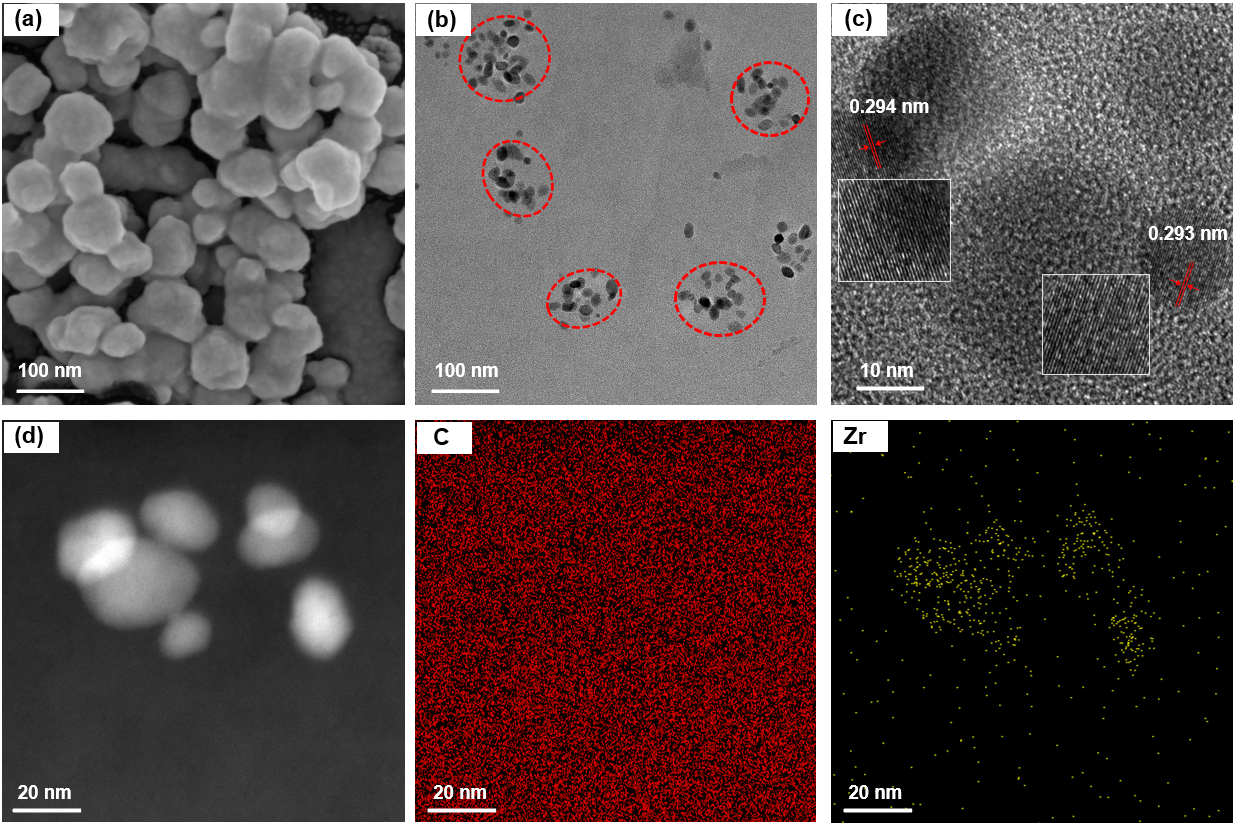


# Figure S12. (a) SEM image of UiO-66; (b) TEM and (c) HRTEM images of PI/UiO66-5-550 MMCMS membrane; (d) HAADF-STEM image of PI/UiO66-5-550 MMCMS membrane and corresponding EDS mappings.


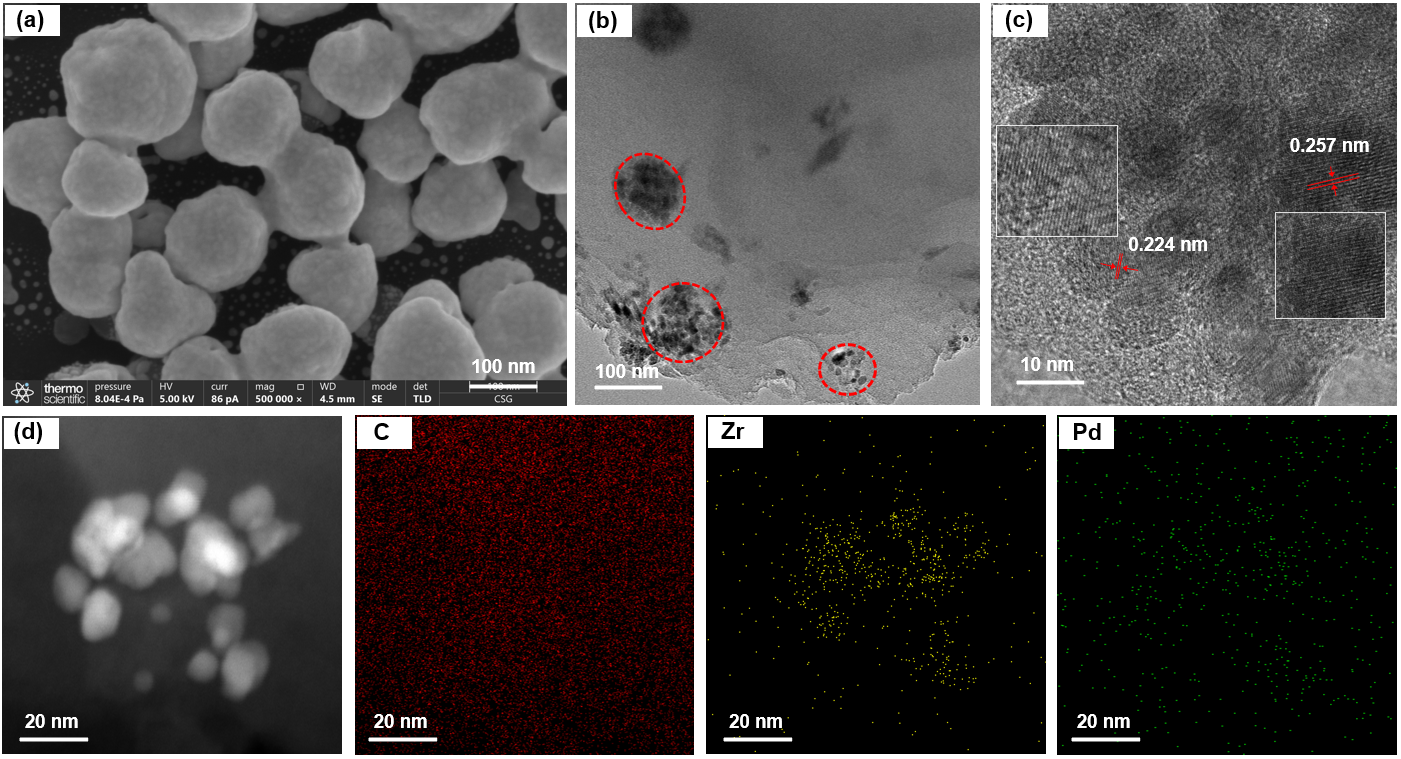


# Figure S13. (a) SEM image of Pd/UiO-66; (b) TEM and (c) HRTEM images of PI/Pd-UiO66-5-550 MMCMS membrane; (d) HAADF-STEM image of PI/Pd-UiO66-5-550 membrane and corresponding EDS mappings.


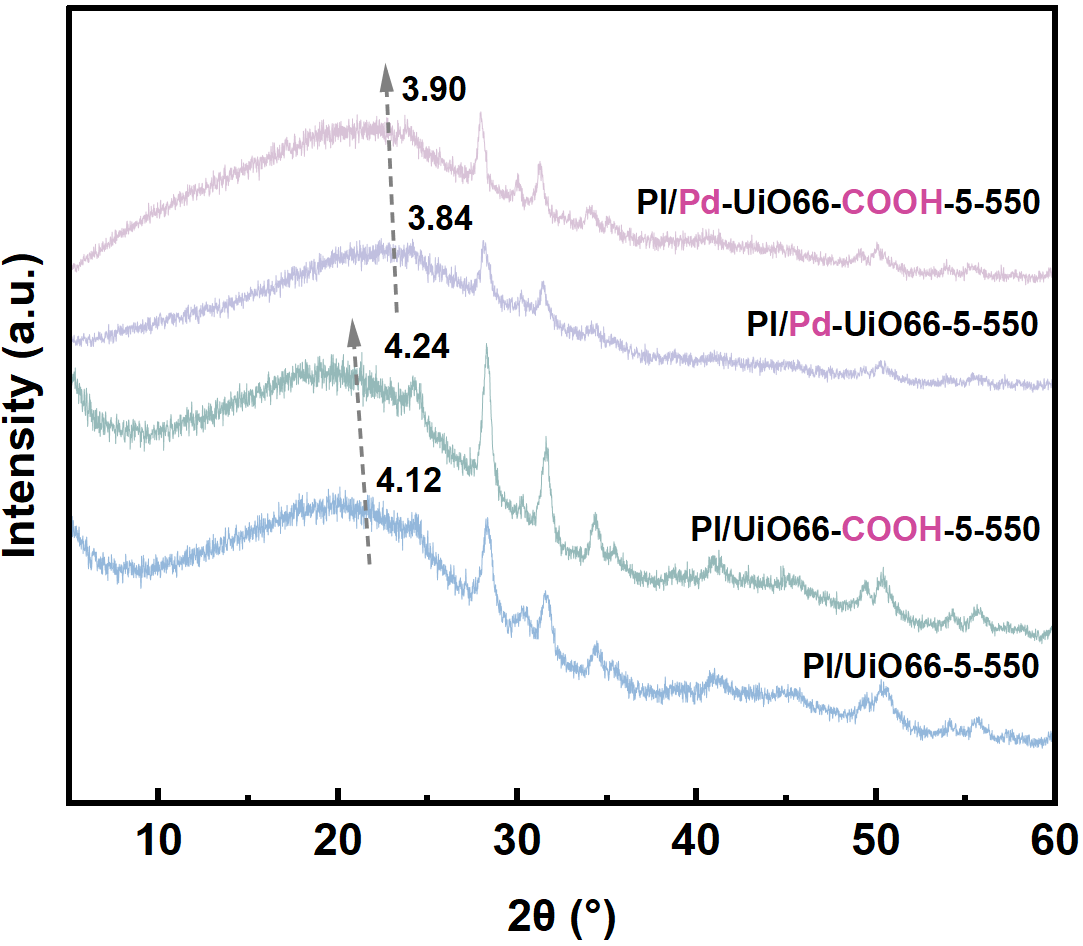


# Figure S14. XRD patterns of PI/UiO66-5-550, PI/UiO66-COOH-5-550, PI/Pd-UiO66-5-550 and PI/Pd-UiO66-COOH-5-550 MMCMS membranes.


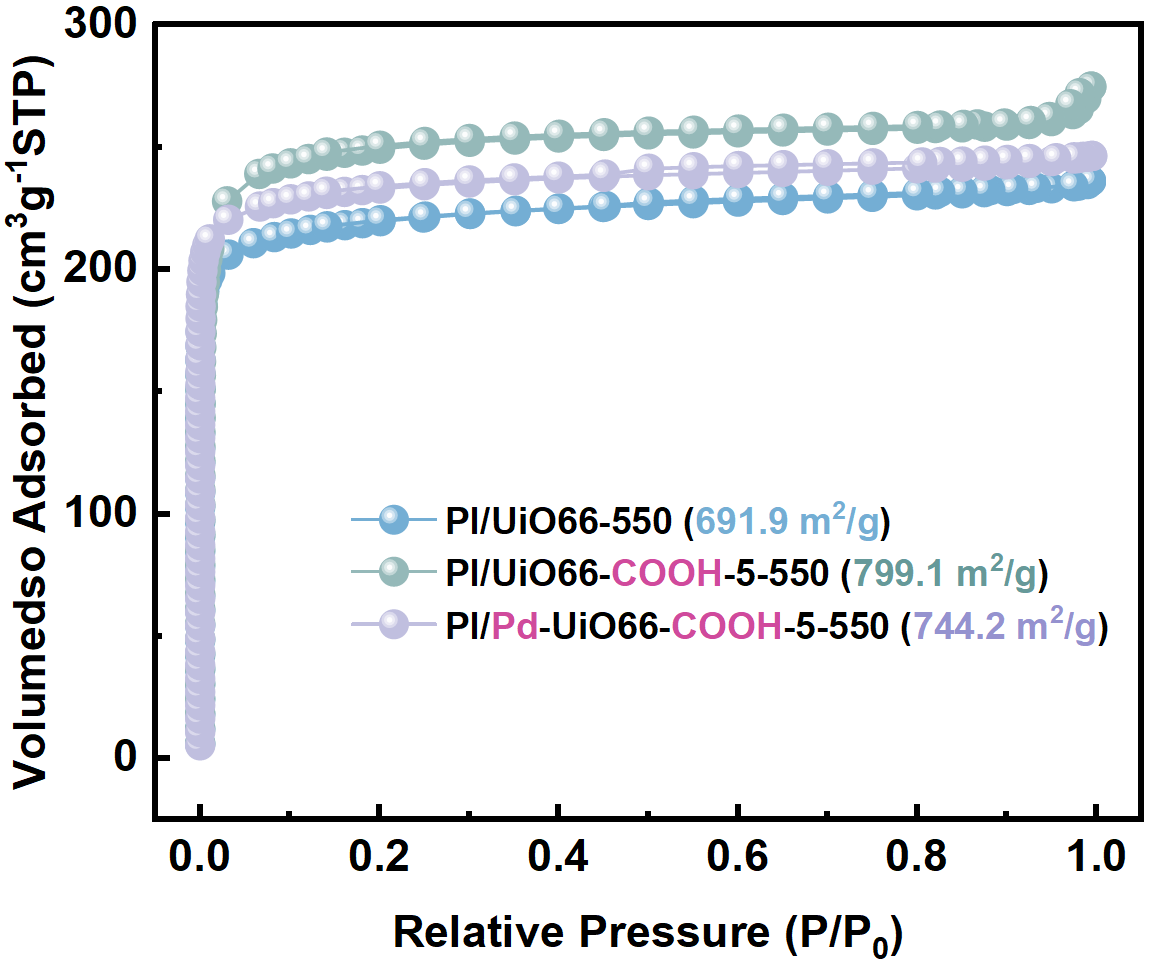


# Figure S15. N_2_ adsorption/desorption isotherms of PI/UiO66-5-550, PI/UiO66-COOH-5-550, and PI/Pd-UiO66-COOH-5-550 MMCMS membranes.


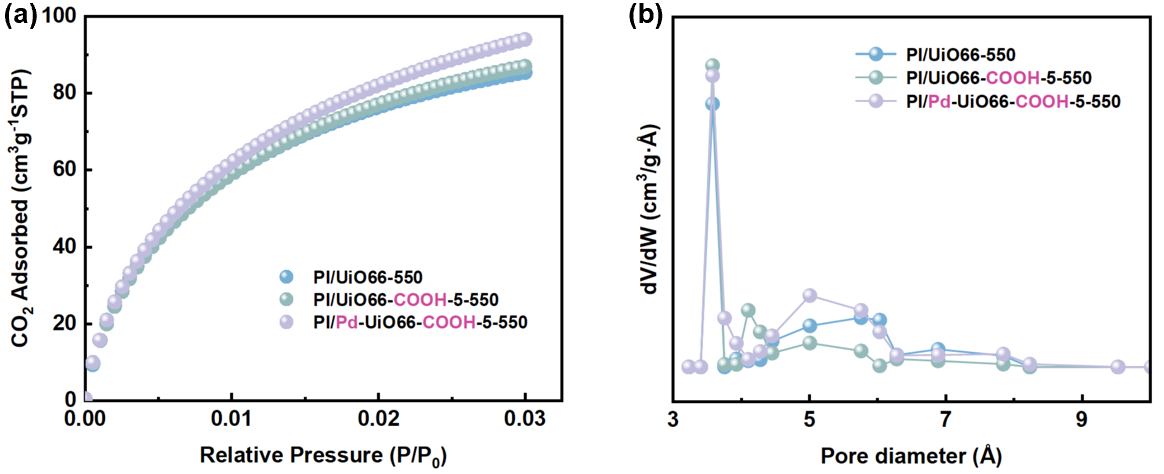


# Figure S16. (a) CO_2_ sorption isotherms measured at 0 °C and (b) pore size distribution of PI/UiO66-5-550, PI/UiO66-COOH-5-550 and PI/Pd-UiO66-COOH-5-550 MMCMS membranes.


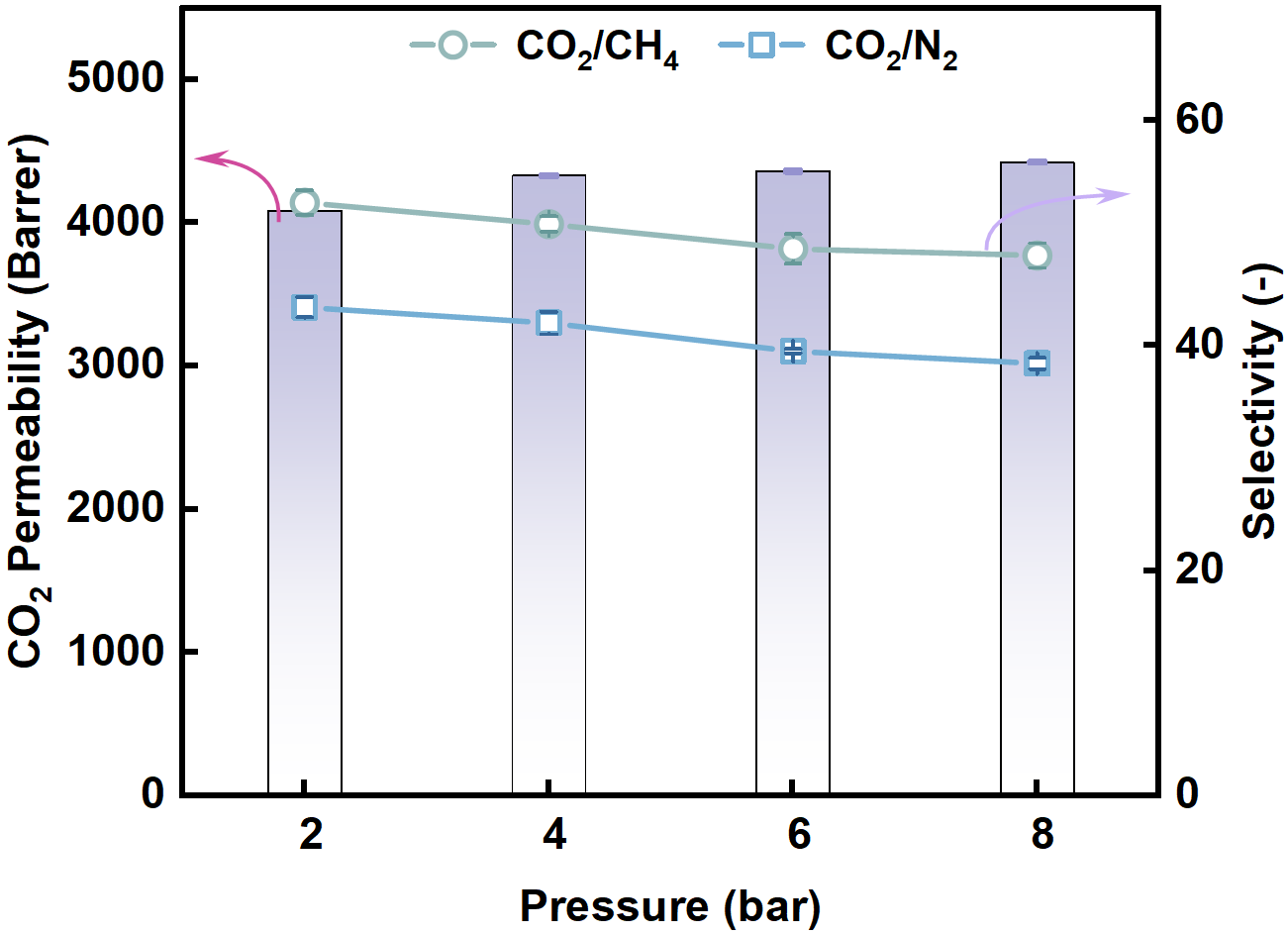


# Figure S17. The effect of feed pressure on CO_2_ separation of PI/Pd-UiO66-COOH-5-550 MMCMS membrane (gas permeation test carried out at 25 °C).


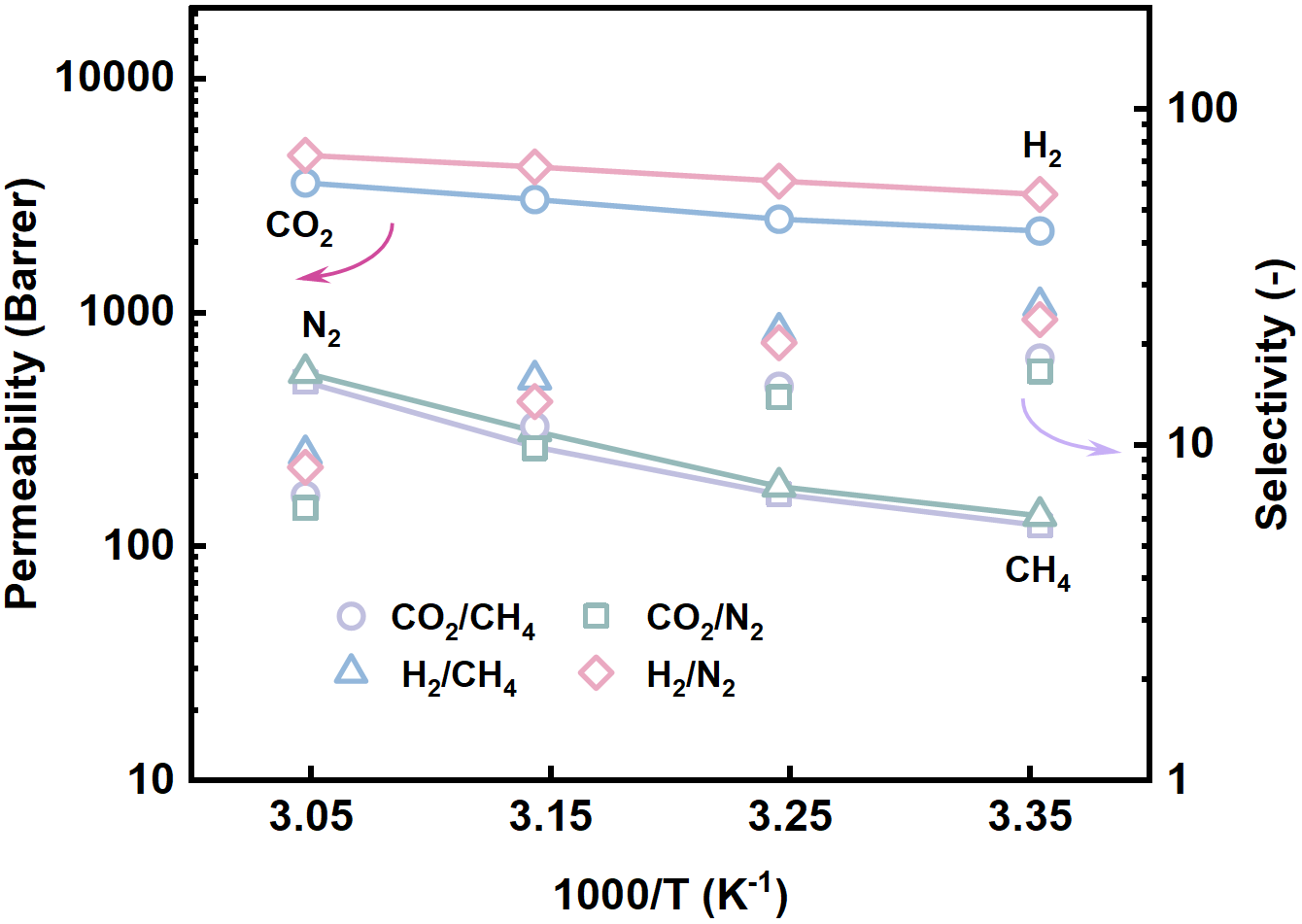


# Figure S18. The effect of test temperature on gas separation of PI-550 CMS membrane (tested at a feed pressure of 2 bar).


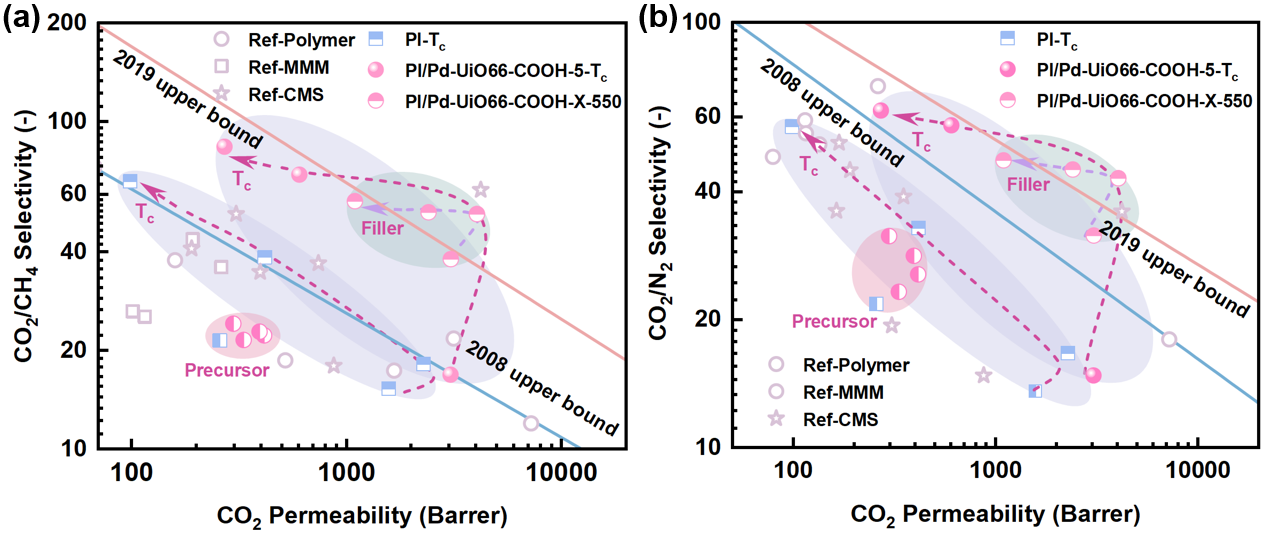


# Figure S19. (a) CO_2_/CH_4_ and (b) CO_2_/N_2_ separation performances of PI/Pd-UiO66-COOH-X precursor membranes and their derived MMCMS membranes compared to the Robeson upper bound.


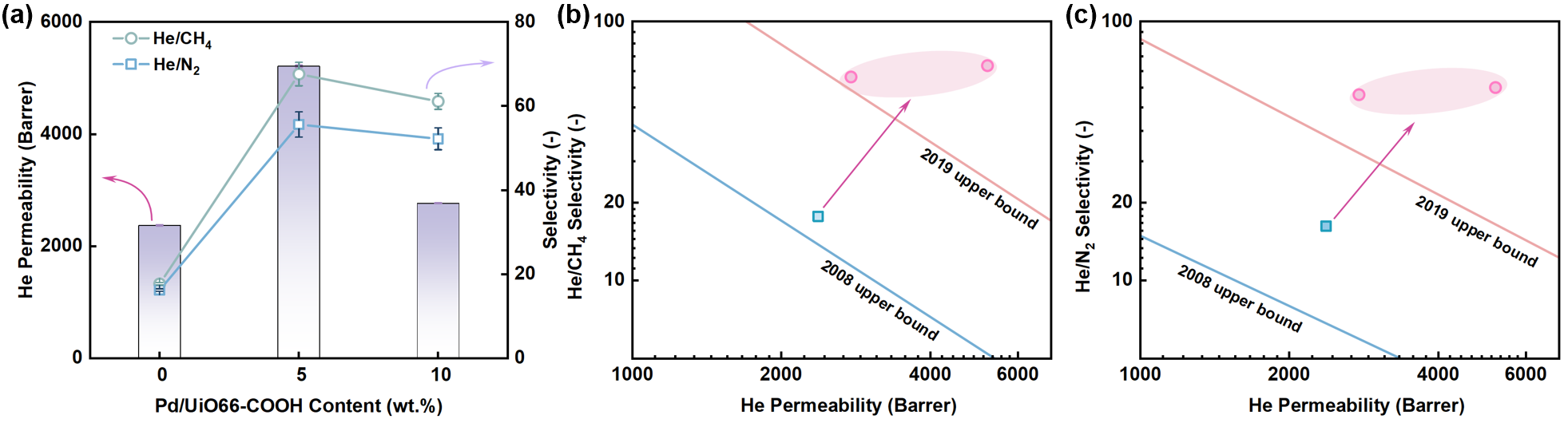


# Figure S20. (a) He permeability and selectivity of PI/Pd-UiO66-COOH-X-550 MMCMS membranes; (b) He/CH_4_ and (c) He/N_2_ separation performances of PI/Pd-UiO66-COOH-X-550 MMCMS membranes compared to the Robeson upper bound.


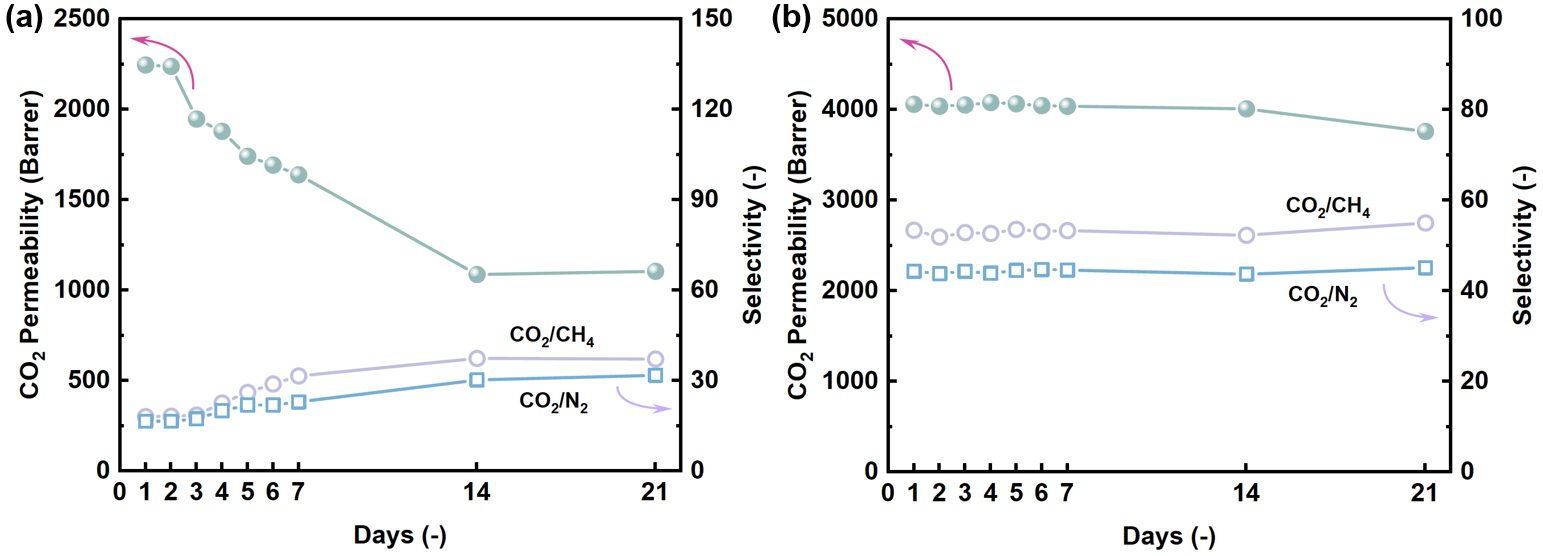


**Figure S21.** Long-term stability of (a)PI-550 CMS membrane and (b) PI/Pd-UiO66-COOH-5-550 MMCMS membrane for CO_2_ separation (tested at a feed pressure of 2 bar at 25 °C).


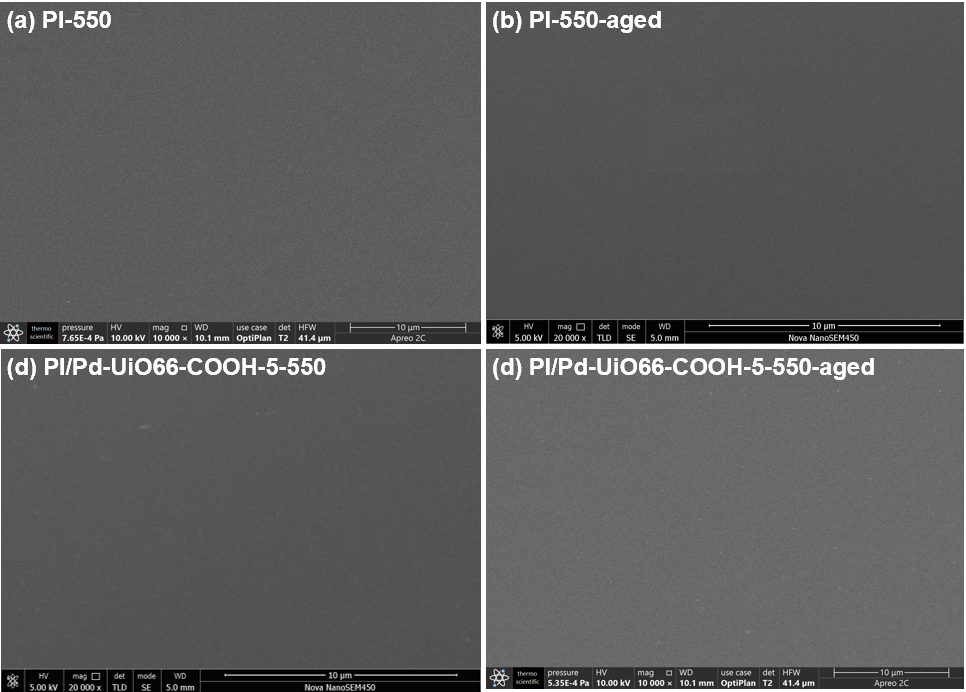


# Figure S22. SEM surface images of PI-550 CMS and PI/Pd-UiO66-COOH-X-550 MMCMS membranes before and after aging.


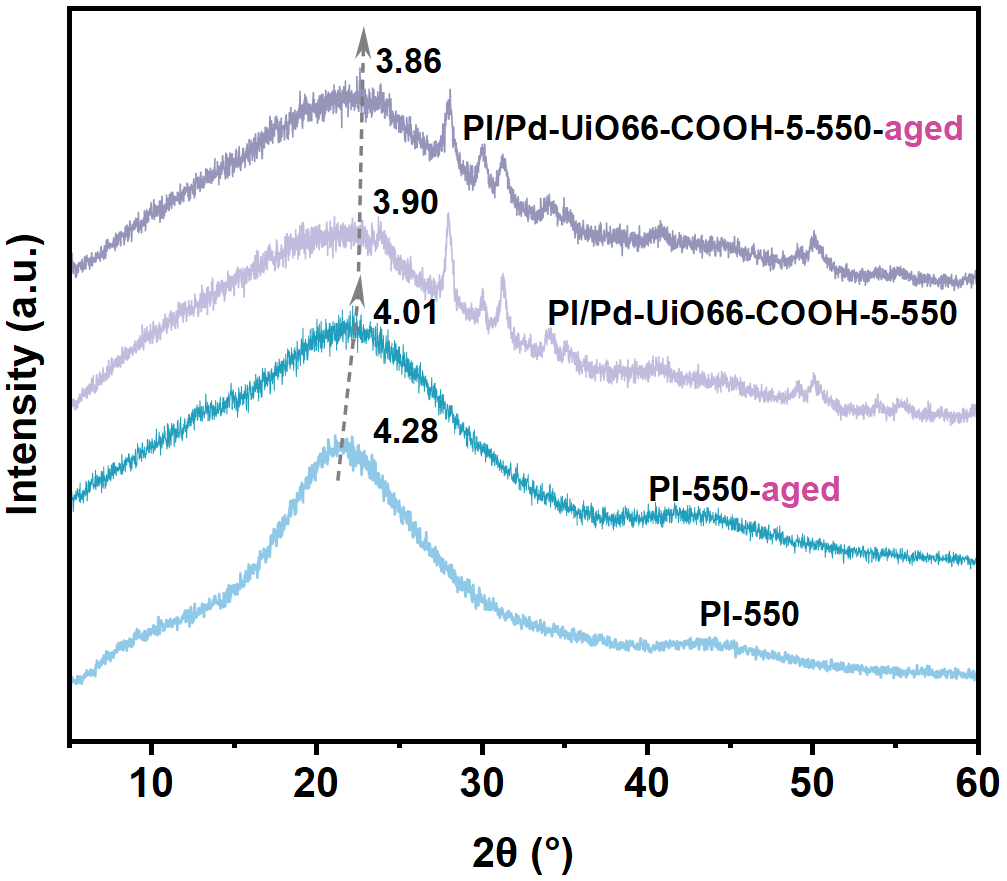


# Figure S23. XRD patterns of PI-550 CMS and PI/Pd-UiO66-COOH-X-550 MMCMS membranes before and after aging.


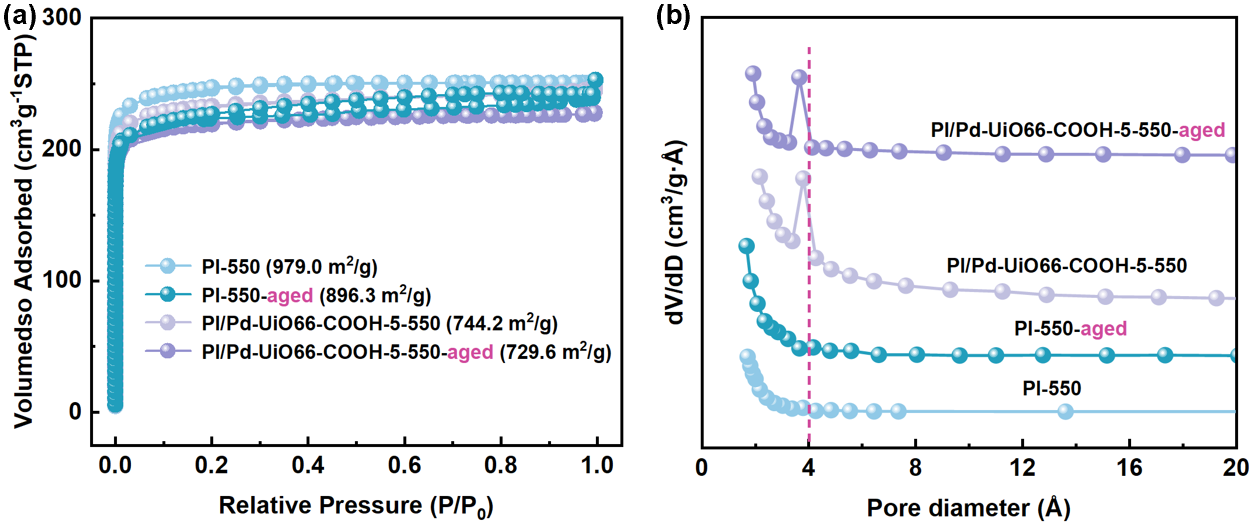


# Figure S24. (a) N_2_ adsorption/desorption isotherms and (b) pore size distribution curves of PI-550 CMS and PI/Pd-UiO66-COOH-X-550 MMCMS membranes before and after aging.


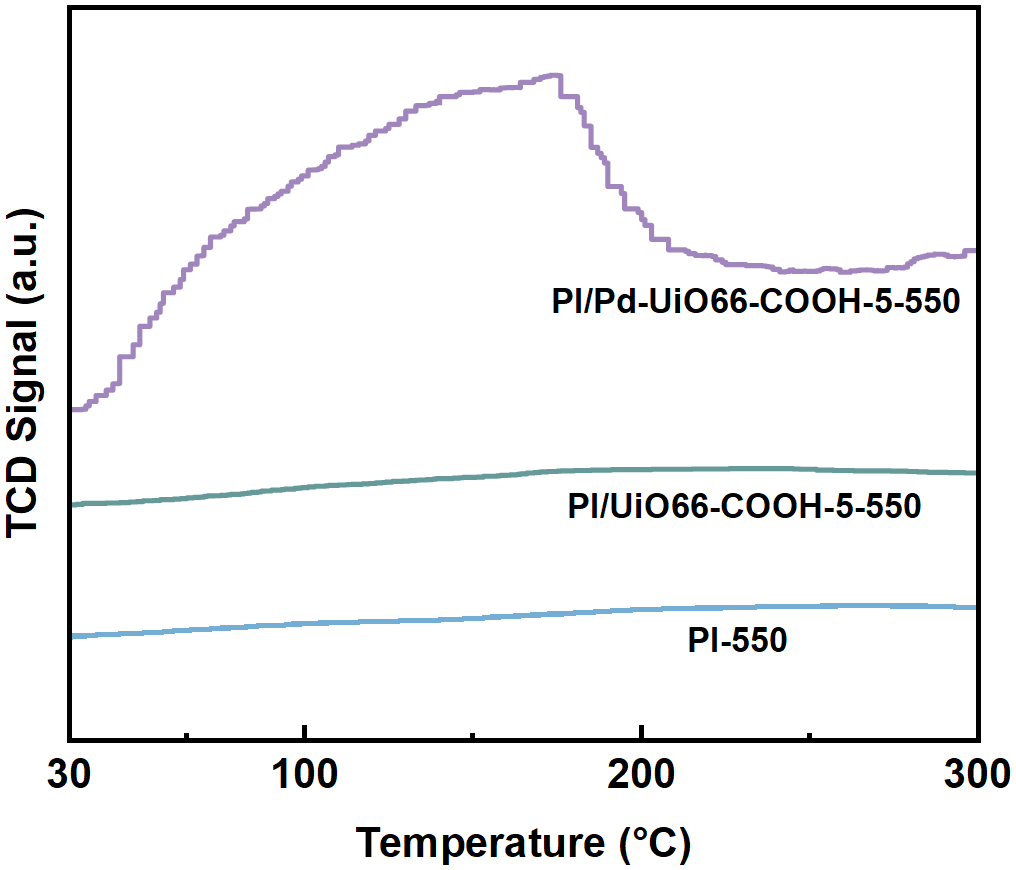


# Figure S25. H2-TPD profiles of PI-550 CMS, PI/UiO66-COOH-5-550 and PI/Pd-UiO66-COOH-5-550 MMCMS membranes.

# Table S1 Metal loadings (ICP) of various samples.

| **Materials** | **Pd loading (wt.%)** |
| --- | --- |
| UiO-66 | - |
| Pd/UiO-66 | 1.4 |
| UiO-66-COOH | - |
| Pd/UiO-66-COOH | 1.5 |
| UiO-66-(COOH)_2_ | - |
| Pd/UiO-66-(COOH)_2_ | 1.5 |

The data was obtained in our previous work^[1]^.

# Table S2 d-spacing of various precursor membranes from XRD patterns.

| **Membrane** | **2θ_1_ (°)** | **2θ_2_ (°)** | **d_1_-spacing (Å)** | **d_2_-spacing (Å)** |
| --- | --- | --- | --- | --- |
| PI | 16.70 | 25.62 | 5.30 | 3.47 |
| PI/Pd-UiO66-COOH-3 | 17.01 | 25.92 | 5.21 | 3.43 |
| PI/Pd-UiO66-COOH-5 | 17.27 | 26.33 | 5.13 | 3.38 |
| PI/Pd-UiO66-COOH-10 | 17.43 | 26.56 | 5.08 | 3.35 |
| PI/Pd-UiO66-COOH-20 | 17.61 | 26.84 | 5.03 | 3.32 |

# Table S3. Peak assignment in the FTIR results.

| **Wavenumber (cm^-1^)** | **Peak assignment** |
| --- | --- |
| 3000-2840 | C-H vibrations of -CH_3_ group |
| 1789 | symmetric stretching vibrations of the C=O bond |
| 1720 | asymmetric stretching vibrations of the C=O bond |
| 1375 | C-N-C stretching vibration |
| 1120-1050 | C-F bond of -CF_3_ group |
| 721 | OC-N-CO imide ring band deformation |

# Table S4 I_G_/I_D_ values in the Raman results from pristine CMS and MMCMS membranes.

| **Membranes** | **I_G_/I_D_ value** |
| --- | --- |
| PI-550 | 1.04 |
| PI/Pd-UiO66-COOH-3-550 | 1.12 |
| PI/Pd-UiO66-COOH-5-550 | 1.13 |
| PI/Pd-UiO66-COOH-10-550 | 1.16 |
| PI/Pd-UiO66-COOH-20-550 | 1.18 |

# Table S5 Textural properties of various materials.

| **Materials** | **S_BET_ (m^2^/g)^a^** | **TPV (cm^3^/g)^b^** |
| --- | --- | --- |
| UiO-66-COOH^c^ | 319.0 | 0.17 |
| Pd/UiO-66-COOH^c^ | 70.0 | 0.09 |
| PI-550 | 979.0 | 0.39 |
| PI/UiO66-5-550 | 691.9 | 0.29 |
| PI/UiO66-COOH-5-550 | 799.1 | 0.42 |
| PI/Pd-UiO66-COOH-5-550 | 744.2 | 0.38 |

^a^BET surface area (S_BET_), ^b^Total pore volume (TPV), ^c^Data was obtained in our previous work.^[1]^

# Table S6 Single-gas separation performances of this work.

| **Membranes** | $\boldsymbol{P}_{\boldsymbol{H}_{\boldsymbol{2}}}$  **(Barrer)** | $\boldsymbol{P}_{\boldsymbol{CO}_{\boldsymbol{2}}}$  **(Barrer)** | $\boldsymbol{\alpha}_{\boldsymbol{H}_{\boldsymbol{2}}\boldsymbol{/}\boldsymbol{CH}_{\boldsymbol{4}}}$**(-)** | $\boldsymbol{\alpha}_{\boldsymbol{H}_{\boldsymbol{2}}\boldsymbol{/}\boldsymbol{N}_{\boldsymbol{2}}}$**(-)** | $\boldsymbol{\alpha}_{\boldsymbol{CO}_{\boldsymbol{2}}\boldsymbol{/}\boldsymbol{CH}_{\boldsymbol{4}}}$**(-)** | $\boldsymbol{\alpha}_{\boldsymbol{CO}_{\boldsymbol{2}}\boldsymbol{/}\boldsymbol{N}_{\boldsymbol{2}}}$**(-)** |
| --- | --- | --- | --- | --- | --- | --- |
| PI | 135.4 | 257.2 | 11.5 | 11.3 | 21.8 | 21.5 |
| PI/Pd-UiO66-COOH-3 | 156.4 | 331.3 | 11.0 | 10.2 | 23.3 | 21.6 |
| PI/Pd-UiO66-COOH-5 | 183.1 | 412.8 | 11.4 | 9.9 | 25.6 | 22.3 |
| PI/Pd-UiO66-COOH-10 | 176.4 | 392.7 | 12.7 | 10.3 | 28.3 | 22.9 |
| PI/Pd-UiO66-COOH-20 | 139.5 | 296.5 | 14.8 | 11.4 | 31.5 | 24.2 |
| PI/Pd-UiO66-COOH-3-550 | 5818.6 | 3049.6 | 72.7 | 60.4 | 38.1 | 31.6 |
| PI/Pd-UiO66-COOH-5-550 | 9134.6 | 4033.4 | 118.5 | 97.5 | 52.3 | 43.1 |
| PI/Pd-UiO66-COOH-10-550 | 7455.1 | 2400.4 | 164.7 | 140.5 | 53.0 | 45.2 |
| PI/Pd-UiO66-COOH-20-550 | 4980.0 | 1090.8 | 261.5 | 216.7 | 57.3 | 47.5 |
| PI-450 | 2079.7 | 1570.2 | 20.2 | 18.0 | 15.3 | 13.6 |
| PI-550 | 3191.3 | 2274.5 | 25.6 | 23.4 | 18.2 | 16.7 |
| PI-650 | 918.9 | 415.7 | 85.3 | 72.8 | 38.6 | 32.9 |
| PI-750 | 219.5 | 98.5 | 146.7 | 127.6 | 65.8 | 57.0 |
| PI/Pd-UiO66-COOH-5-450 | 5111.4 | 3041.3 | 28.3 | 24.8 | 16.9 | 14.8 |
| PI/Pd-UiO66-COOH-5-550 | 9134.6 | 4033.4 | 118.5 | 97.5 | 52.3 | 43.1 |
| PI/Pd-UiO66-COOH-5-650 | 3737.9 | 602.3 | 428.0 | 355.9 | 69.0 | 57.4 |
| PI/Pd-UiO66-COOH-5-750 | 1547.4 | 270.1 | 619.0 | 458.5 | 84.0 | 62.2 |
| PI/UiO66-5-550 | 5132.6 | 2839.5 | 108.7 | 90.2 | 48.2 | 40.9 |
| PI/UiO66-COOH-5-550 | 7811.8 | 3662.1 | 118.5 | 97.5 | 53.0 | 43.5 |
| PI/Pd-UiO66-5-550 | 6795.5 | 3124.4 | 134.1 | 106.7 | 50.0 | 41.5 |
| PI/Pd-UiO66-COOH-5-550 | 9134.6 | 4033.4 | 87.1 | 74.0 | 52.3 | 43.1 |
| PI/Pd-UiO66-(COOH)_2_-5-550 | 8327.9 | 3551.7 | 114.1 | 93.6 | 57.2 | 45.5 |

# Table S7 Activation energy of permeation for different gases in PI-550 CMS membrane and PI/Pd-UiO66-COOH-5-550 MMCMS membranes.

| **Membranes** | **E_P_ (kJ/mol)** | | | |
| --- | --- | --- | --- | --- |
|  | **H_2_** | **CO_2_** | **N_2_** | **CH_4_** |
| PI-550 | 10.64 | 13.35 | 34.35 | 41.99 |
| PI/Pd-UiO66-COOH-5-550 | 4.75 | 15.26 | 27.95 | 33.57 |

# Table S8 Comparison of H_2_ separation performance of polymer membranes, MMMs and CMS membranes derived from different precursors.

|  | **Membrane** | $\boldsymbol{P}_{\boldsymbol{H}_{\boldsymbol{2}}}$  **(Barrer)** | $\boldsymbol{\alpha}_{\boldsymbol{H}_{\boldsymbol{2}}\boldsymbol{/}\boldsymbol{CH}_{\boldsymbol{4}}}$**(-)** | $\boldsymbol{\alpha}_{\boldsymbol{H}_{\boldsymbol{2}}\boldsymbol{/}\boldsymbol{N}_{\boldsymbol{2}}}$**(-)** | **Ref** |
| --- | --- | --- | --- | --- | --- |
| Polymer  membranes | PIM-1 | 3380 | 5.9 | 8.5 | ^[4]^ |
|  | PIM-NH_2_ | 1450 | 6.8 | 10.8 | ^[4]^ |
|  | DFTTB | 5468 | 38.0 | 50.2 | ^[5]^ |
|  | CTTB | 5257 | 26.3 | 37.6 | ^[6]^ |
|  | 6FDA-HTB | 167 | 181.0 | - | ^[7]^ |
|  | CTB1-DMN | 1295 | 13.5 | 17.0 | ^[8]^ |
|  | 6FDA-BI | 33 | 278.2 | - | ^[9]^ |
| Mixed matrix  membranes | HPI/10%ZIF8 | 300 | 68.2 | 37.8 | ^[10]^ |
|  | APBO/30%ZIF8 | 212 | 55.4 | - | ^[11]^ |
|  | 6FDA-DAM/10% Z67@Z8 (L) | 662 | 22.9 | 17.4 | ^[12]^ |
|  | 6FDA-BI/20%ZIF-8 | 79 | 223.9 | - | ^[9]^ |
|  | COOH-PI/5%NH_2_-UiO-66 | 848 | 25.2 | - | ^[13]^ |
| CMS membranes | P84/ZCC-800 | 88 | 10.4 | - | ^[14]^ |
|  | TB-PI-650 | 6552 | 96.0 | 56.0 | ^[15]^ |
|  | PEI-Al_2_O_3_-600 | 537 | 197.6 | - | ^[16]^ |
|  | cellulose-3.5-Pd-700 | 87 | 1169.0 | 366.7 | ^[17]^ |
|  | PI-siloxane (2:1)-600 | 7618 | 32.1 | 31.6 | ^[18]^ |
|  | BAHPPF-ODPA-600 | 3838 | - | 147.6 | ^[19]^ |
|  | PI/UiO66-5-550 | 5133 | 87.1 | 74.0 | This work |
|  | PI/UiO66-COOH-5-550 | 7812 | 114.1 | 93.6 |  |
|  | PI/Pd-UiO66-5-550 | 6796 | 108.7 | 90.2 |  |
|  | PI/Pd-UiO66-COOH-5-550 | 9135 | 118.5 | 97.5 |  |
|  | PI/Pd-UiO66-(COOH)_2_-5-550 | 8328 | 134.1 | 106.7 |  |

# Table S9 Comparison of CO_2_ separation performance of polymer membranes, MMMs and CMS membranes derived from different precursors.

|  | **Membrane** | $\boldsymbol{P}_{\boldsymbol{CO}_{\boldsymbol{2}}}$  **(Barrer)** | $\boldsymbol{\alpha}_{\boldsymbol{CO}_{\boldsymbol{2}}\boldsymbol{/}\boldsymbol{CH}_{\boldsymbol{4}}}$**(-)** | $\boldsymbol{\alpha}_{\boldsymbol{CO}_{\boldsymbol{2}}\boldsymbol{/}\boldsymbol{N}_{\boldsymbol{2}}}$**(-)** | **Ref** |
| --- | --- | --- | --- | --- | --- |
| Polymer  membranes | PIM-1 | 7200 | 12.0 | 18.0 | ^[4]^ |
|  | PIM-NH_2_ | 840 | 4.0 | 6.3 | ^[4]^ |
|  | DFTTB | 3146 | 21.8 | - | ^[5]^ |
|  | 6FDA-HTB | 67 | 73.0 | - | ^[7]^ |
|  | CTB1-DMN | 1661 | 17.4 | - | ^[8]^ |
|  | 6FDA-DAM | 518 | 18.7 | - | ^[20]^ |
|  | Matrimid | 10 | 34.5 | - | ^[20]^ |
|  | 6FDA-DAM:DABA (3:2) | 159 | 37.8 | - | ^[21]^ |
|  | Pebax 1657 | 79 | - | 48.4 | ^[22]^ |
|  | Cellulose acetate | 13 | 7.6 | 6.2 | ^[23]^ |
|  | Polysulfone | 6 | 26.9 | 28.4 | ^[24]^ |
| Mixed matrix  membranes | HPI/10%ZIF8 | 192 | 43.8 | - | ^[10]^ |
|  | PIM-1/10% ZIF-67 | 13256 | 7.4 | 9.3 | ^[25]^ |
|  | APBO/30%ZIF8 | 101 | 26.4 | - | ^[11]^ |
|  | 6FDA-BI/20%ZIF8 | 20 | 57.9 | 25.9 | ^[9]^ |
|  | Pebax1657/8%ZIF8 | 261 | 36.0 | 71.0 | ^[26]^ |
|  | Pebax1657/10%ZIF8-NH_2_ | 114 | - | 59.0 | ^[22]^ |
|  | Pebax1657/20%UiO66 | 115 | 25.4 | 55.0 | ^[27]^ |
|  | Pebax1657/15%UiO66-(OH)_2_ | 134 | - | 51.8 | ^[28]^ |
| CMS membranes | TB-PI | 4200 | 62.0 | 36.0 | ^[15]^ |
|  | P84 | 738 | 37.0 | - | ^[29]^ |
|  | PMDA/pPDA | 396 | 34.8 | - | ^[30]^ |
|  | BTDA-ODA | 350 | - | 39.0 | ^[31]^ |
|  | Matrimid | 871 | 18.0 | 14.8 | ^[32]^ |
|  | PBI-UIP-R | 168 | - | 52.2 | ^[33]^ |
|  | PBI-P84 HT | 163 | - | 36.1 | ^[33]^ |
|  | PBI/Matrimid | 306 | 52.3 | 19.4 | ^[34]^ |
|  | Cellulose | 190 | 41.0 | 45.0 | ^[35]^ |
|  | PEI | 53 | - | 17.6 | ^[36]^ |
|  | PI/UiO66-5 | 2840 | 48.2 | 40.9 | This work |
|  | PI/UiO66-COOH-5 | 3662 | 53.0 | 43.5 |  |
|  | PI/Pd-UiO66-5 | 3124 | 50.0 | 41.5 |  |
|  | PI/Pd-UiO66-COOH-5 | 4033 | 52.3 | 43.1 |  |
|  | PI/Pd-UiO66-(COOH)_2_-5 | 3552 | 57.2 | 45.5 |  |

**Reference**

[1] R. Yangcheng, J. Li, J. He, Y. Zheng, H. Yu, C. Chen, J. Wang, *Small* **2024**, *20*, 2309821.

[2] M. Deng, J. Wei, W. Du, Z. Qin, Z. Zhang, L. Yang, L. Yao, W. Jiang, B. Tang, X. Ma, Z. Dai, *ACS Applied Materials & Interfaces* **2024**, *16*, 44927-44937.

[3] J. Wei, M. Yulei, Q. Zikang, D. Jing, S. Roman, L. Nanwen, F. Lu, L. Zhikao, D. Liyuan, Y. Shouliang, S. R. J., Z. and Dai, *Polymer Reviews*, 1-58.

[4] K. Mizrahi Rodriguez, S. Lin, A. X. Wu, G. Han, J. J. Teesdale, C. M. Doherty, Z. P. Smith, *Angewandte Chemie International Edition* **2021**, *60*, 6593-6599.

[5] X. Ma, Z. Zhu, W. Shi, W. Ji, J. Li, Y. Wang, I. Pinnau, *Journal of Materials Chemistry A* **2021**, *9*, 5404-5414.

[6] Z. Zhu, J. Zhu, J. Li, X. Ma, *Macromolecules* **2020**, *53*, 1573-1584.

[7] X. Ma, M. Abdulhamid, X. Miao, I. Pinnau, *Macromolecules* **2017**, *50*, 9569-9576.

[8] X. Ma, M. A. Abdulhamid, I. Pinnau, *Macromolecules* **2017**, *50*, 5850-5857.

[9] Y. Fan, H. Yu, S. Xu, Q. Shen, H. Ye, N. Li, *Journal of Membrane Science* **2020**, *597*, 117775.

[10] J. S. Kim, S. J. Moon, H. H. Wang, S. Kim, Y. M. Lee, *Journal of Membrane Science* **2019**, *582*, 381-390.

[11] S. Japip, S. Erifin, T.-S. Chung, *Separation and Purification Technology* **2019**, *212*, 965-973.

[12] S. H. Yuan, A. P. Isfahani, T. Yamamoto, A. Muchtar, C. Y. Wu, G. Huang, Y. C. You, E. Sivaniah, B. K. Chang, B. Ghalei, *Small Methods* **2020**, *4*, 2000021.

[13] Z. Wang, Y. Tian, W. Fang, B. B. Shrestha, M. Huang, J. Jin, *ACS Applied Materials & Interfaces* **2021**, *13*, 3166-3174.

[14] N. Widiastuti, A. R. Widyanto, I. S. Caralin, T. Gunawan, R. Wijiyanti, W. N. Wan Salleh, A. F. Ismail, M. Nomura, K. Suzuki, *ACS Omega* **2021**, *6*, 15637-15650.

[15] Z. Wang, H. Ren, S. Zhang, F. Zhang, J. Jin, *ChemSusChem* **2018**, *11*, 916-923.

[16] H.-H. Tseng, C.-T. Wang, G.-L. Zhuang, P. Uchytil, J. Reznickova, K. Setnickova, *Journal of Membrane Science* **2016**, *510*, 391-404.

[17] G. Zhao, K. Wang, C. Fang, Y. Wang, D. Wang, Z. Song, L. Lei, Z. Xu, *AIChE Journal* **2024**, *70*, e18413.

[18] H. Li, B. Zhao, Y. Yu, Y. Liu, *Separation and Purification Technology* **2023**, *320*, 124168.

[19] M. Hou, L. Li, Z. He, R. Xu, Y. Lu, T. Wang, *Carbon* **2023**, *205*, 194-206.

[20] J. E. Bachman, J. R. Long, *Energy & Environmental Science* **2016**, *9*, 2031-2036.

[21] H. Eguchi, D. J. Kim, W. J. Koros, *Polymer* **2015**, *58*, 121-129.

[22] R. Ding, Z. Li, Y. Dai, X. Li, X. Ruan, J. Gao, W. Zheng, G. He, *Sep. Purif. Technol.* **2022**, *298*, 121594.

[23] Z. Hu, H. Zhang, X.-F. Zhang, M. Jia, J. Yao, *J. Membr. Sci.* **2022**, *662*, 120996.

[24] Z. Tahir, M. Aslam, M. A. Gilani, M. R. Bilad, M. W. Anjum, L.-P. Zhu, A. L. Khan, *Sep. Purif. Technol.* **2019**, *224*, 524-533.

[25] J. Han, L. Bai, H. Jiang, S. Zeng, B. Yang, Y. Bai, X. Zhang, *Ind. Eng. Chem. Res.* **2021**, *60*, 593-603.

[26] A. Jomekian, B. Bazooyar, R. M. Behbahani, T. Mohammadi, A. Kargari, *Journal of Membrane Science* **2017**, *524*, 652-662.

[27] Z. Iqbal, Z. Shamair, M. Usman, M. A. Gilani, M. Yasin, S. Saqib, A. L. Khan, *Chemosphere* **2022**, *303*, 135122.

[28] Z. Yang, Y. Ying, Y. Pu, D. Wang, H. Yang, D. Zhao, *Ind. Eng. Chem. Res.* **2022**, *61*, 7626-7633.

[29] S. T. Pei, T. S. Chung, L. Ye, R. Wang, *Carbon* **2004**, *42*, 3123-3131.

[30] W.L. Qiu, J.E. Leisen, Z.Y. Liu, W.Y. Quan, W. J. Koros, *Angewandte Chemie International Edition* **2021**, *60*, 22322- 22331.

[31] Y. Kim, H. Park, Y. Lee, *Journal of Membrane Science* **2005**, *255*, 265-273.

[32] J. Xiao, Y. Dai, T. S. Chung, M. D. Guiver, *Macromolecules* **2005**, *38*, 10042-10049.

[33] S. S. Hosseini, M. R. Omidkhah, A. Zarringhalam Moghaddam, V. Pirouzfar, W. B. Krantz, N. R. Tan, *Separation and Purification Technology* **2014**, *122*, 278-289.

[34] S. S. Hosseini, T. S. Chung, *Journal of Membrane Science* **2009**, *328*, 174-185.

[35] J. A. Lie, M.-B. Hägg, *Carbon* **2005**, *43*, 2600-2607.

[36] P. S. Rao, M.-Y. Wey, H.-H. Tseng, I. A. Kumar, T.-H. Weng, *Microporous and Mesoporous Materials* **2008**, *113*, 499-510.
